# Resolving the evolutionary duality of marine symbionts: redefining the genus *Endozoicomonas* and proposing *Neoendozoicomonas* gen. nov

**DOI:** 10.1093/ismeco/ycag123

**Published:** 2026-05-13

**Authors:** Flúvio Modolon, Alessandro N Garritano, Philip Hugenholtz, Christian R Voolstra, Tina Keller-Costa, Rodrigo Costa, Torsten Thomas, Raquel S Peixoto

**Affiliations:** Department of Ecology, Environment and Geoscience, Umeå University, Umeå, Västerbotten 90187, Sweden; School of Life and Environmental Sciences, University of Sydney, Sydney, New South Wales 2006, Australia; Centre for Marine Science and Innovation & School of Biological, Earth and Environmental Sciences, The University of New South Wales, Sydney, New South Wales 2052, Australia; Australian Centre for Ecogenomics, School of Chemistry and Molecular Biosciences, The University of Queensland, Brisbane, Queensland 4072, Australia; Department of Biology, University of Konstanz, Konstanz, Baden-Württemberg 78457, Germany; Institute for Bioengineering and Biosciences (iBB) and Institute for Health and Bioeconomy (i4HB), Instituto Superior Técnico, University of Lisbon, Lisbon, 1049 001, Portugal; Department of Bioengineering, Instituto Superior Técnico, University of Lisbon, Lisbon, 1049 001, Portugal; Institute for Bioengineering and Biosciences (iBB) and Institute for Health and Bioeconomy (i4HB), Instituto Superior Técnico, University of Lisbon, Lisbon, 1049 001, Portugal; Department of Bioengineering, Instituto Superior Técnico, University of Lisbon, Lisbon, 1049 001, Portugal; Centre for Marine Science and Innovation & School of Biological, Earth and Environmental Sciences, The University of New South Wales, Sydney, New South Wales 2052, Australia; Biological and Environmental Science and Engineering Division, King Abdullah University of Science and Technology, Thuwal, Makkah Province 23955, Saudi Arabia

**Keywords:** *Endozoicomonas*, *Neoendozoicomonas*, *Endonucleibacter*, *Sororendozoicomonas*, cpAAI, taxonomy, coral symbiosis, comparative genomics, SeqCode

## Abstract

Members of the bacterial family *Endozoicomonadaceae* are ubiquitous marine symbionts associated with diverse hosts, including corals, sponges, molluscs, and fishes. Despite their ecological pervasiveness, inhabiting hosts in deep-sea vents and sunlit coral reefs, their taxonomy remains inconsistent, largely due to reliance on 16S rRNA gene phylogenies that fail to fully resolve evolutionary relationships. Here, we integrate phylogenomics, comparative genomics, and overall genome relatedness indices across 63 high-quality genomes to re-define genus boundaries within the *Endozoicomonadaceae* family. Among the metrics, the core-proteome average amino acid identity proved the most robust for genus-level resolution, with a proposed identity cutoff of 76%, aligning with phylogenomic structure and maintaining taxonomic robustness across the family. Phylogenomic reconstructions further revealed that the taxon *Endozoicomonas* is paraphyletic, forming two well-supported but distinct clades. This prompts a taxonomic revision, dividing *Endozoicomonas* into *Endozoicomonas sensu stricto* and *Neoendozoicomonas* gen. nov., with *Neoendozoicomonas montiporae* designated as the type species. Furthermore, the sister clade to *Endozoicomonas sensu stricto*—comprising the genera *Endonucleibacter* and *Sororendozoicomonas*—is characterized by genomic streamlining, including reduced genome size, lower GC content, reduced number of orthogroups, and potential functional divergences consistent with host specialization. Functional annotations highlighted secretion and conjugation systems as key differentiators between genera, emphasizing potential distinct host-interaction strategies. This genome-based framework refines the taxonomy of the *Endozoicomonadaceae*, provides additional criteria for genus delimitation, and strengthens the evolutionary and ecological interpretation of these widespread marine bacterial symbionts.

## Introduction

The genus *Endozoicomonas* in the gammaproteobacterial family *Endozoicomonadaceae* represents one of the most widespread and functionally diverse groups of marine symbionts. Members of this genus are consistently found across a broad range of hosts—including corals, sponges, echinoderms, molluscs, and fishes—where they can represent dominant microbiome members [1–7]. Their metabolic versatility and ecological ubiquity suggest central roles in marine symbioses and ecosystem functioning. For example, recent multi-omics and experimental studies have revealed that *Endozoicomonas* spp. symbionts contribute to multiple aspects of host physiology, including nutrient provisioning (e.g. amino acid biosynthesis, vitamin B₁₂ and ammonia production), sulfur and nitrogen cycling, and modulation of host immune and stress responses [3, 4, 6, 8, 9]. Emerging evidence also points towards a complex ecological duality: while many *Endozoicomonas* strains likely act as beneficial symbionts, others have been implicated in disease outbreaks affecting fishes and marine invertebrates [4, 10–12].

A deep phylogenetic split within the genus *Endozoicomonas* has been consistently observed in the literature and is commonly referred to as “clade A” and “clade B” [9, 13–18]. Gotze et al. [16] studied isolates corresponding to the two major *Endozoicomonas* clades*,* each exhibiting distinct genomic signatures and inferred symbiotic strategies: one clade appears to specialize in degrading holobiont-derived carbon and lipid compounds (including steroid hormones), while the other is enriched in type VI secretion systems and phosphorus acquisition genes, potentially mediating host-microbe signalling and stress adaptation. da Silva et al. [9] further provided a comprehensive genomic perspective on the *Endozoicomonadaceae* family, revealing extensive metabolic versatility across marine hosts and evidence for a mutualism–parasitism continuum. Microscopy evidence further indicates that the two clades may adopt distinct strategies for establishing associations within host cells [17]. Together, these findings underscore that evolutionary diversification within the *Endozoicomonadaceae* family has substantial ecological and functional consequences.

Despite the clear separation within the *Endozoicomonas* genus, the clade-based taxonomy of *Endozoicomonadaceae* remains poorly resolved. Taxonomy solely based on 16S rRNA gene phylogeny and a limited set of phenotypic traits, has proven insufficient to discern the genomic and ecological diversity of bacterial species [19], and may result in inconsistent classifications and uncertain taxonomic boundaries. Ecological inferences might further be impacted by lineages with closely related 16S rRNA gene sequences with different host specificity, virulence, or metabolic capacity.

Genome-level metrics, such as average nucleotide identity (ANI), average amino acid identity (AAI), and core-proteome phylogenomics can improve resolution of species- and genus-level boundaries not afforded by single-gene approaches [20–24]. However, a genome-based taxonomy for members of the *Endozoicomonadaceae* family has not been formally proposed. The absence of such definition limits comparative studies and complicates the integration of this taxon’s data into larger microbial ecology and symbiosis frameworks. In addition, it perpetuates taxonomic inconsistencies and the ambiguous use of “clade” terminology, which conflates taxa with distinct ecological roles. This simplification not only obscures evolutionary history but may also affect ecological surveys of microbial communities, as demonstrated by the resolution of distinct ecotypes in abundant marine lineages [25–27] and hamper the identification of beneficial strains for microbial therapies or coral probiotics [28, 29].

This study establishes genome-based thresholds to resolve the taxonomy of the *Endozoicomonadaceae* family and redefines the genus *Endozoicomonas*. By integrating phylogenomic, overall genome-relatedness indices (OGRIs) and functional enrichment analyses across 63 high-quality genomes, we identify consistent genomic and evolutionary boundaries that justify the division of *Endozoicomonas* into two distinct genera: *Endozoicomonas sensu stricto* and *Neoendozoicomonas* gen. nov. We also evidenced *Endonucleibacter* and *Sororendozoicomonas* as two genera that, although forming sister lineages within a monophyletic clade, fall below the cpAAI threshold proposed here for genus delimitation. This refined taxonomy provides robust, genome-informed criteria for genus-level classification within the *Endozoicomonadaceae*, facilitating accurate lineage identification and improved communication and reproducibility in symbiosis research.

## Material and methods

### Genomic data

A preliminary search for genomes assigned to the genus *Endozoicomonas* and related taxa of the family *Endozoicomonadaceae* was performed on the Genome Taxonomy Database (GTDB, Release 226, v2.4.1) [30] in November 2025. Resultant accession IDs were cross-referenced against the NCBI Assembly database to remove redundant assemblies and to identify additional publicly available genomes. Quality filtering retained assemblies meeting the thresholds of >90% completeness and <5% contamination (according to CheckM2 [31]). Discordant assemblies exhibiting atypical genomic content (e.g., excessive fragmentation or GenBank anomaly flags) were excluded. The curated dataset comprised 57 genomes assigned to the family *Endozoicomonadaceae*, supplemented by six outgroup genomes. The outgroups were defined by selecting type species from the closest related families according to previous observation [32]. All accessions IDs are catalogued in Supplementary Data S1.

### Phylogenomic inferences using multiple single-copy genes

A phylogenomic dataset was constructed using 138 single-copy orthologous clusters, retrieved via OrthoFinder (v2.5.5) [33]. Multiple sequence alignment was performed with MAFFT v7.526 [34], followed by trimming with trimAl using a gap threshold of 50% (−gt 0.50) [35]. The best-fit model of evolution was defined using ModelTest-NG (v0.1.7) [36]. Maximum likelihood (ML) phylogeny was inferred using RAxML-ng v1.2.2 [37] under the defined LG + I + G4 model, with 10 randomized parsimony starting trees. Node support was assessed via 1000 non-parametric bootstrap replicates. Final topology was visualized and annotated in iTOL v7 [38].

### Phylogenetic inferences using 16S rRNA gene sequences

16S rRNA gene sequences for the available genera assigned to the *Endozoicomonadaceae* family were downloaded from the List of Prokaryotic names with Standing in Nomenclature (https://lpsn.dsmz.de/, accessed on August 13, 2025). A total of 18 sequences from the *Endozoicomonadaceae* family were retrieved (one per species), encompassing the genera *Parendozoicomonas* (2 sequences/species), *Endonucleibacter* (2 sequences/species), *Kistimonas* (3), and the current *Endozoicomonas* (11), besides two outgroups. The partial 16S rRNA gene sequences were aligned with MAFFT v7.526 [34, 38] using default parameters, followed by trimming with TrimAl v1.4.1 [35] (−gt 0.5). The final alignments (1433 positions) served as input for the iqtree (v3) software [39] with the automated evolutionary model selection and 1000 standard bootstrap replicates. Final topology was visualized in iTOL v7 [38].

### Estimating the OGRIs

We developed a complete pipeline for OGRI estimations, which is available at https://github.com/modolon/phylo_endozoi. Briefly, pairwise genome similarity was determined using the Average Nucleotide Identity (ANI) method implemented in the fastANI tool (v1.34) [40]. The AAI was determined using EzAAI [41]. The core-proteome average amino acid identity (cpAAI) values were computed in the software EzAAI, using only the single-copy core orthologs, extracted using OrthoFinder (v2.5.5) [33] as described above. To compute the “percentage of conserved protein” (POCP) index, the conserved protein pairs between genomes were firstly identified through reciprocal BLASTP alignments [42] using the standard thresholds [22]: E-value <1e−5, >40% sequence identity, and >50% query coverage. Each genome served alternately as the query to mitigate paralog-related discrepancies. The POCP was calculated as [(C1 + C2)/(T1 + T2)] × 100%, where C1 and C2 denote conserved proteins in each genome, and T1 and T2 represent total encoded proteins. POCP values (theoretical range: 0%–100%) reflect proteomic similarity between strains. The cutoff of 50% is indicated for genus delimitation [22].

### Comparative genome analyses

We developed a custom Python script to analyse orthogroup distributions among the studied genera. The script first parsed a genome–genus mapping file to assign each genome to its proposed genus and then filtered the Orthogroups.tsv file generated by OrthoFinder [33] to retain only the target genomes. For each orthogroup, the script identified whether it was exclusive to a single genus, shared between two genera, or conserved across all genera, and further classified exclusive orthogroups according to their conservation level within a genus (100%, ≥75%, ≥50% of genomes). Singleton genes were extracted from the Orthogroups_UnassignedGenes.tsv file and counted per genus. Finally, summary statistics, including total orthogroups, genus-specific exclusivity and conservation, shared orthogroups, core orthogroups, and singleton counts, were written to an output report for comparative genomic interpretation.

Functional annotation of sequences and functional enrichment analysis were performed using MetaCerberus [43] with the KOFam_prokaryote hidden Markov model database to identify KEGG Orthology (KO) assignments. The resulting KO contingency table was used for downstream differential abundance distribution and pathway enrichment analyses, which provides conservative significance estimates based on count-based models [44]. Briefly, MetaCerberus performs differential abundance analysis of KO terms using DESeq2 (v1.48.2) [45] and the differential abundance testing was conducted using the Wald test to obtain maximum likelihood estimate Log₂ fold changes, p-values, and adjusted p-values (Benjamini-Hochberg FDR correction). Differentially abundant KO terms were identified using adjusted p-value (padj) < 0.05 and Log₂ fold change >2. Volcano plots were generated using the EnhancedVolcano package (v.1.26) to visualize the relationship between fold change magnitude and statistical significance across all KO terms.

## Results

### Phylogenomic analysis of the family *Endozoicomonadaceae*

**Figure 1:**
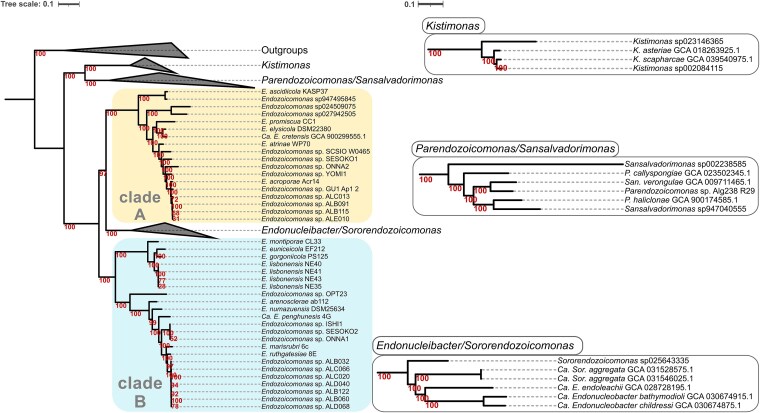
Phylogenomic tree of the family *Endozoicomonadaceae*. The taxonomy of each genome in the tree corresponds to the previous recognized nomenclature as it was at the moment of download. Bootstrap support values are indicated by red numerals. The branch corresponding to what was conventionally referred to as “clade A” and “clade B” are indicated by background boxes.

Phylogenomic reconstruction based on 139 concatenated, conserved genes revealed a deep bifurcation in the presently defined genus *Endozoicomonas,* partitioning into two distinct, well-supported groups (Fig. 1). One branch comprised the five described species *Endozoicomonas elysicola, E. promiscua, E. ascidiicola, E. atrinae, E. acroporae, Candidatus* E. cretensis and other lineages without species description. The second branch consisted of the seven described species *Endozoicomonas montiporae, E. gorgoniicola, E. marisrubri, E. euniceicola, E. lisbonensis, E. arenoscleare, E. numazensis, and Candidatus* E. ruthgatesiae among other lineages without a species epithet. These two groups have been previously referred to as “clade A” and “clade B”, respectively [9, 13–17]. Although these designations do not constitute formal taxonomic ranks, they are used here to facilitate interpretation of the phylogenomic results and allow comparison with earlier work. A third branch containing genomes assigned to the genera *Endonucleibacter* (syn. *Endonucleobacter*) and *Sororendozoicomonas* formed a well-supported monophyletic clade (100% bootstrap). This clade formed a sister group to clade A with 97% bootstrap support, rendering the taxon *Endozoicomonas* paraphyletic. The clade comprising members of the genera *Parendozoicomonas* and *Sansalvadorimonas* formed a monophyletic group, as a sister group to the *Kistimonas* genus (Fig. 1), with strong bootstrap support (100).

The phylogenetic tree based on the 16S rRNA gene also supports paraphyly of the *Endozoicomonas* genus, forming two well-supported clades (bootstrap support values >90%): one containing *E. elysicola, E. ascidiicola, E. atrinae, E. coralli*, and *E. acroporae*, and a second containing *E. gorgoniicola, E. euniceicola, E. lisbonensis, E. montiporae, E. numazensis,* and *E. arenosclerae* (Fig. S1). The species content of both clades is congruent with the phylogenomic tree (Fig. 1), where the first clade represents clade A and the second clade B. However, the 16S rRNA gene-based phylogeny differed from the phylogenomics tree in that: (i) *Endonucleibacter* and *Kistimonas* form a well-supported clade (99.8% bootstrap); and (ii) the *Endonucleibacter*-*Kistimonas* clade share a common ancestor with clade B (Fig. S1).

### Comparison of OGRIs supports reclassification of genus boundaries in the *Endozoicomonadaceae* family

**Figure 2:**
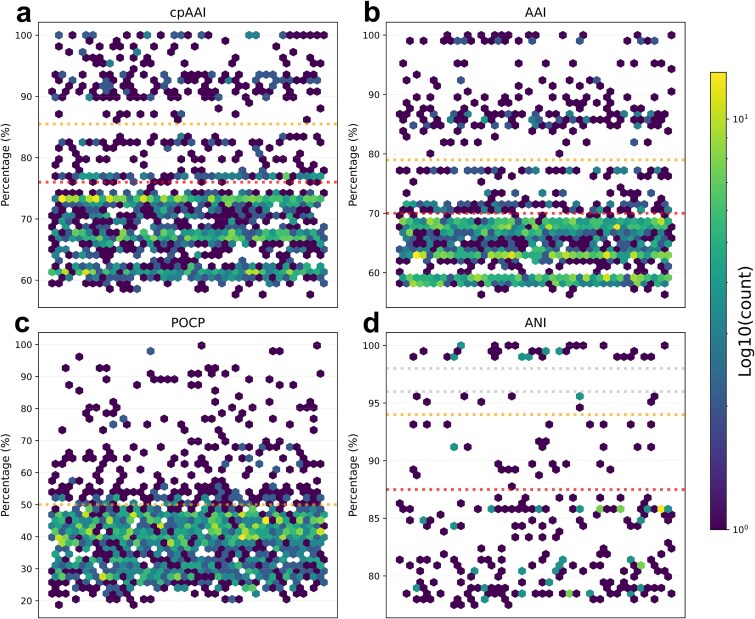
Density distributions of pairwise genome relatedness indices. Hexbin plots compare values for (a) core-proteome cpAAI, (b) AAI, (c) POCPs, and (d) ANI. Hexagon colors represent the density of pairwise comparisons (*Log*_10_ scale), ranging from dark purple (low density) to bright yellow (high density). Breakpoints are marked by orange dashed lines for the higher values (cpAAI = 85.5%, AAI = 79%, ANI = 94, POCP = 50%) and red dashed lines for the lower values (cpAAI = 76%, AAI = 70%, ANI = 87.5). Silver dashed lines on ANI indicate the interval between 96% and 98%. Values below ≈ 77% of ANI were automatically discarded due to low confident inference.

Comparative analysis of OGRIs revealed distinct patterns in their resolution and applicability for genus-level delimitation within the family *Endozoicomonadaceae*. Among the evaluated metrics, the cpAAI metric showed two breakpoints at 85.5 and 76% (Fig. 2a). The AAI showed one clear breakpoint at 79% and a second, more diffuse one at 70%. The POCP exhibited substantial variance compared to ANI, AAI, and cpAAI (Fig. 2a, b, c, d). Although a 50% threshold has been proposed as a genus-level cutoff for POCP [22], no clear breakpoint was observed around this value in our dataset (Fig. 2), indicating that this metric is unsuitable for genus delimitation within the *Endozoicomonadaceae* family. Lastly, ANI values revealed breakpoints between 96%–98%, at 94% and 87.5% (Fig. 2); However, even the lowest value plotted (77%) failed to delineate most existing genera within *Endozoicomonadaceae* (see Fig. S2), clustering only the most closely related genomes. The complete cpAAI heatmap, including values below the investigated cutoffs, is available in Fig. S5. For the ANI, AAI, and POCP heatmaps, see Figs. S2, S4, and  S6, respectively.

**Figure 3:**
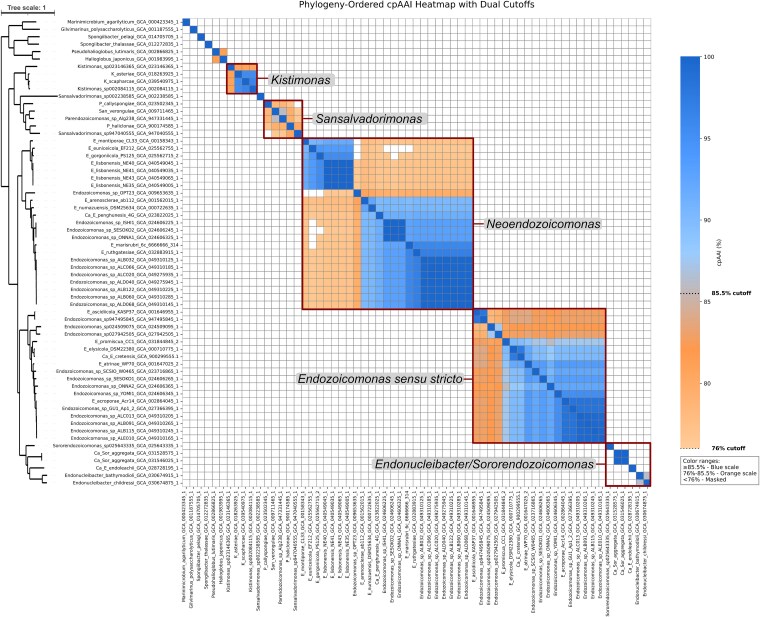
Heatmap for cpAAI showing dual potential cutoffs for cluster boundaries, 76 and 85.5%. The five clades representing the genera in the *Endozoicomonadaceae* family are highlighted by red rectangles. The heatmap was ordered according to the tree topology (left side).

The OGRI analysis corroborates the phylogenomic evidence regarding the divergence of *Endozoicomonas* clades A and B (Fig. 3). Consequently, we propose splitting *Endozoicomonas* into two distinct genera: *Endozoicomonas sensu stricto* (corresponding to clade A) and *Neoendozoicomonas* gen. nov. (corresponding to clade B).

We propose a cpAAI cutoff of 76% to define genus boundaries, provided that their monophyly is supported in the phylogenomic tree (Fig. 1), as this threshold enabled the preservation of most currently accepted genera within the *Endozoicomonadaceae* family. A similar clustering pattern was observed with the 70% cutoff for AAI (Figs. S3 and S4), which could also be used to distinguish between genera within the *Endozoicomonadaceae* family. Within the *Neoendozoicomonas* genus, a clear internal divergence into two sub-clades was observed in the phylogenomic tree (Fig. 1), which is aligned with previous results [9, 18]. However, the high genomic similarity between these sub-branches—with internal cpAAI values consistently exceeding 76% (Fig. 3)—supports their classification within a single genus. Although *Endonucleibacter* and *Sororendozoicomonas* form a clade, they remain distinct under current OGRI thresholds; thus, we suggest maintaining their separate status, with ultimate taxonomic revisions pending on further genomic data. Furthermore, our analysis also indicates that *Sansalvadorimonas* and *Parendozoicomonas* constitute a single genus clade. This classification is established by their internal cpAAI values, which consistently exceed the 76% threshold (Fig. 3), and is further reinforced by their robust monophyly in the phylogenomic reconstruction (Fig. 1). Consequently, we propose the union of these two taxa under the genus *Sansalvadorimonas* and the transfer of all species previously assigned to *Parendozoicomonas* to this genus.

### Genomic signatures differentiating *Endozoicomonas* and *Neoendozoicomonas*

The observed divergences in the phylogenomic tree and genus-level cutoffs justify the reclassification of *Endozoicomonas* into *Endozoicomonas sensu stricto* (clade A) and *Neoendozoicomonas* gen. nov. (clade B) (for a full description see below). To compare these groups and avoid potential noise from distantly related lineages, we excluded other genera within the family *Endozoicomonadaceae*, but included the most closely related taxa of the *Endonucleibacter* cluster. The comparative analysis identified 13 731 orthogroups across the three groups. The group of *Endonucleibacter/Sororendozoicomonas* (n = 6 genomes) contained 984 exclusive orthogroups (total count of orthogroups present in all genomes, without distinguishing core and accessory orthogroups of the given genus), including 14 conserved orthogroups (core orthogroups of the given genus) in all six genomes (Supplementary Table S1) and 88 conserved orthogroups in at least half. *Endozoicomonas sensu stricto* (18 genomes) had 2945 exclusive orthogroups, with 13 conserved in all 18 genomes and 341 shared by 50% of its genomes. *Neoendozoicomonas* (22 genomes) showed the highest number of exclusive orthogroups (3794), including 14 conserved in all genomes and 326 conserved in 50% of the genome set assigned to this proposed genus.

Pairwise comparisons revealed substantial differences in shared orthogroups. *Endozoicomonas sensu stricto* and *Neoendozoicomonas* shared 16.88% (2318) of a total of 13 731 orthogroups, while *Endonucleibacter/Sororendozoicomonas* showed limited orthogroup overlap with *Endozoicomonas sensu stricto* (334) and *Neoendozoicomonas* (371). This reduced overlap between *Endonucleibacter/Sororendozoicomonas* and its sister groups could be explained by the reduced genome size and small number of available genomes. A core set of 2985 orthogroups was conserved across the three groups. Singleton gene counts varied considerably between genera, with *Endozoicomonas sensu stricto* containing the most (4879 total, average per genome = 271), followed by *Neoendozoicomonas* (3504 total, average per genome = 159.30) and *Endonucleibacter/Sororendozoicomonas* (1441 total, average per genome = 240). These patterns suggest varying degrees of genomic diversification among the genera, with *Endozoicomonas sensu stricto* exhibiting the greatest intra-genus genomic variation, followed by *Neoendozoicomonas.* The complete detailed orthogroup distributions and conservation patterns are provided in Supplementary Table S1.

The average genome size (Table 1) for *Endonucleibacter/Sororendozoicomonas* is 3.89 Mbp (± 0.74), which is substantially reduced in comparison to *Endozoicomonas sensu stricto* (5.60 bp ± 0.96) and *Neoendozoicomonas* (6.22 Mbp ± 0.85). The GC content is also reduced for the genomes of *Endonucleibacter/Sororendozoicomonas* (Table 1), with an average of 43.42% (± 3.09), in comparison to 48.26% (± 2.48) for *Endozoicomonas sensu stricto* and 47.98% (± 0.91) for *Neoendozoicomonas*. Taken together, these genomic differences indicate genome streamlining in *Endonucleibacter/Sororendozoicomonas*, supporting its evolutionary distinction from its sister taxa.

**Table 1:** Average genome size and GC content by genus in the family *Endozoicomonadaceae*.

Genus	Average size (Mpb)	Average GC (%)
*Endonucleibacter/Sororendozoicomonas*	3.89 ± 0.74	43.42 ± 3.09
*Endozoicomonas sensu stricto*	5.60 ± 0.96	48.26 ± 2.48
*Neoendozoicomonas* gen. nov.	6.22 ± 0.85	47.92 ± 0.91
*Kistimonas*	4.21 ± 1.36	49.73 ± 3.76
*Sansalvadorimonas* comb. nov*.*	4.52 ± 0.67	50.72 ± 3.21

### Functional profiling shows genetic signatures differentiating *Endozoicomonas* and *Neoendozoicomonas*

**Figure 4:**
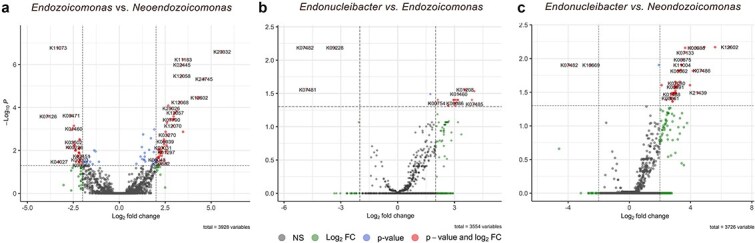
Differential functional abundance between *Endozoicomonadaceae* genera. Volcano plots showing differential abundance analysis of KOs across three pairwise comparisons: (a) *Endozoicomonas vs. Neoendozoicomonas*, (b) *Endonucleibacter/Sororendozoicomonas vs. Endozoicomonas*, and (c) *Endonucleibacter/Sororendozoicomonas vs. Neoendozoicomonas*. The x-axis represents the Log₂ fold change (FC) in abundance, while the y-axis shows the –Log₁₀ *P*-value, indicating statistical significance. Points are coloured according to significance: grey (NS): non-significant; green (Log₂ FC): significant fold change only; blue (*P*-value): significant *P*-value only; light red (*P*-value and Log₂ FC): significant for both criteria. Dashed horizontal lines indicate the *P*-value significance threshold, and vertical lines mark Log₂ FC cutoffs.

Functional profiling revealed genes (assigned to KEGG orthologs) distinguishing the genera *Endozoicomonas sensu stricto* and *Neoendozoicomonas*. In total, 38 KO terms were significantly enriched in *Endozoicomonas sensu stricto*, whereas *Neoendozoicomonas* showed enrichment of 58 KO terms (p_adj_-value <0.05, Fig. 4a). The *Neoendozoicomonas* genus was primarily defined by a high abundance of genes associated with the Type IV secretion system and conjugal transfer (Fig. S7). This cluster showed significantly elevated z-scores for a suite of *tra* genes, including *traI, traD, traK, traE, traA, traV, traB*, and *traG*. Additionally, this genus exhibited differential metabolic traits, such as the enrichment of glucokinase (*glk*) for glycolysis, the glycerol-3-phosphate transporter, and *cobS*, a cobaltochelatase involved in porphyrin and vitamin B12 metabolism.

In contrast, the *Endozoicomonas sensu stricto* genomes were characterized by the enrichment of distinct transport and stress response mechanisms, most notably the putrescine transport system (Fig. S7). This genus displayed high relative abundances for the polyamine ABC transporter components *potF, potG, potH*, and *potI*, indicating a potential reliance on scavenging environmental or host-derived polyamines. Furthermore, *Endozoicomonas sensu stricto* genomes showed a higher prevalence of genes for ethanolamine utilization (*eutM*) and the cobalt-zinc-cadmium efflux system, pointing towards metabolic strategies tailored to phospholipid scavenging and heavy metal homeostasis that are largely absent or less pronounced in *Neoendozoicomonas*.

The comparison between *Endozoicomonas sensu stricto* and *Endonucleibacter/Sororendozoicomonas* revealed an enrichment of three KEGG orthologs assigned to mobile genetic elements and specific biosynthetic pathways in the *Endonucleibacter/Sororendozoicomonas* genomes in contrast to *Endozoicomonas* (Figs. 4b, S8). *Endonucleibacter/Sororendozoicomonas* was characterized by high z-scores for multiple transposases (e.g. IS5 and IS30 families, K07481, K07482) and RNA-directed DNA polymerases (*ltrA*). In contrast, *Endozoicomonas sensu stricto* exhibited distinct functional signatures (23 KEGG orthologs), mostly related to biofilm formation (*exoP*) and environmental sensing, including multidrug efflux pumps (*mdlB*) and glycerol-3-phosphate dehydrogenases (*glpA*) (Figs. 4b, S8). Metabolically, *Endozoicomonas sensu stricto* showed specific enrichments for quorum sensing-related toxoflavin biosynthesis (*toxA*) and glutathionylspermidine metabolism, as well as starch metabolism enzymes like cyclomaltodextrinase (*cd*).

When compared to *Neoendozoicomonas, Endonucleibacter/Sororendozoicomonas* again displayed a defining abundance of transposases and replication-associated proteins (2 KEGG orthologs, Figs. 4c, S9). In contrast, *Neoendozoicomonas* possesses distinctive metabolic traits such as nitrate and nitrite reduction (*napA, nirB*, which represent the complete pathway for dissimilatory nitrate reduction to ammonia, or DNRA) and purine metabolism enzymes. The distinction was most pronounced in secretion capabilities; *Neoendozoicomonas* was strongly differentiated by the comprehensive presence of the Type IV secretion system (conjugal transfer genes including *traI, traD, traK, traE*), which was largely absent in *Endonucleibacter/Sororendozoicomonas* group. Furthermore, *Neoendozoicomonas* showed enrichment in genes for fatty acid biosynthesis and iron transport systems (*afuA*).

## Discussion

Our study extends previous findings on the taxonomy and ecology of the *Endozoicomonadaceae* family, thereby providing new insights into its evolutionary relationships and functional diversity. Genome-wide analyses support the division of the genus *Endozoicomonas* into two distinct genera, and we propose the establishment of the new genus *Neoendozoicomonas* gen. nov., for which *Neoendozoicomonas montiporae* [46] is designated as the type species, following the principle of priority as the first described species of this group.

Our phylogenomic analysis revealed the paraphyletic nature of the current *Endozoicomonas* genus, whilst the OGRIs helped delineate a proxy for genus boundaries within the *Endozoicomonadaceae* family. It is important to note, however, that our findings contrast with the monophyletic grouping previously described for *Endozoicomonas* [9, 16, 47, 48], in which the genus *Endonucleibacter* occupies a distinct branch outside the *Endozoicomonas* lineage. Methodological differences have most likely led to previous inconsistent placements of *Endonucleibacter*. Prior studies have employed a wide range of models and support metrics, from the WAG+G4 model used by Shao et al. [48], which yielded moderate bootstrap support (~0.8), to the LG + F + R7 model applied by Götze et al. [16] with support of <0.9. Other analyses reported strong overall support despite unclear methodological foundations, as in Porras et al. [47], who did not specify the evolutionary model, did not include the *Sororendozoicomonas* lineages, and recovered weak resolution for the split between *Endozoicomonas* clades A and B (37.8 SH-aLRT; 63 UFBoot). da Silva et al. [9] used FastTree and Jukes–Cantor+CAT approximation, producing bootstrap values generally above 0.7. In contrast, our phylogenomic reconstruction showed 97% bootstrap support for placing *Endonucleibacter/Sororendozoicomonas* as a sister group to the *Endozoicomonas sensu stricto*. The high bootstrap support recovered in our reconstruction therefore reflects both increased phylogenetic signal and a model better suited to the evolutionary complexity of the *Endozoicomonadaceae*. In addition, this deep split is consistent with the GTDB R226 release, which separates the traditional *Endozoicomonas* into two genus-level clusters (*Endozoicomonas* and *Endozoicomonas*_A).

Indeed, no consensus was found in earlier studies that assessed the phylogenetic relationship among *Endozoicomonadaceae* genera. For example, Maire et al. [9, 49] noted paraphyly of *Kistimonas* and *Parendozoicomonas* (contrasting with both Porras et al. [47] and our study) and also placed *Endonucleibacter/Sororendozoicomonas* as distinct from *Endozoicomonas*. Recent genome-based surveys emphasized the substantial ecological and functional differences within the genus *Endozoicomonas* [9, 16, 17, 47]. A consistent observation across all previous studies has been the presence of a deep split of the *Endozoicomonas* genus into two well-supported groups, widely referred to as clades A and B [6, 7, 9, 16, 17, 47, 49, 50].

The divergent topologies observed between phylogenomic and 16S rRNA gene trees further illustrate the limitations of single-marker approaches for resolving evolutionary relationships within the *Endozoicomonadaceae*. Nevertheless, the 16S rRNA gene tree recovered cohesive clades corresponding to currently recognized genera and separated *Endozoicomonas sensu stricto* from the lineage here proposed as *Neoendozoicomonas* gen. nov., indicating that 16S rRNA genes retain practical utility for genus-level classification.

The genera *Endonucleibacter* and *Sororendozoicomonas* seem to derive from a common ancestor to *Endozoicomonas sensu stricto* with several adaptations. *Endonucleibacter* and *Sororendozoicomonas* exhibit clear signals of genome streamlining relative to *Endozoicomonas sensu stricto* and *Neoendozoicomonas*, characterized by reduced genome size, lower GC content, and markedly fewer exclusive orthogroups and singletons, in accordance with previous observations by Porras et al. [47] and da Silva et al. [9]. This pattern is consistent with evolutionary trajectories observed in other symbiotic or host-associated bacteria that undergo loss of functional gene content as part of obligatory symbiotic specialization (e.g. genome reduction in intracellular symbionts such as *Buchnera aphidicola*) [51]. In contrast, both *Endozoicomonas sensu stricto* and *Neoendozoicomonas* possess larger genomes with variable gene content, likely enabling them to establish associations with a wider range of hosts and possibly, to transition between free-living and host-associated lifestyles [9].

### Functional divergence between *Endozoicomonas sensu stricto* and *Neoendozoicomonas* gen. nov.

The previous classification of *Endozoicomonas* clades A and B as a single genus is understandable, given their overlapping genomic and ecological characteristics, such as comparable G + C content and a broad distribution across marine hosts, particularly cnidarians. Nevertheless, this grouping risks masking important functional heterogeneity, which has been recently explored [9, 16, 17] and provide additional insights to our study. For example, *Endozoicomonas sensu stricto* differs from *Neoendozoicomonas* mainly through a marked enrichment of genes related to conjugative pili and components of secretion and transport systems. Moreover, shifts in the abundance of eukaryotic-like proteins underpin the divergence of *Endozoicomonas sensu stricto* from *Neoendozoicomonas*, with *Endozoicomonas sensu stricto* enriched in diversified ankyrin repeat proteins, whereas WD40 repeats are more abundant in *Neoendozoicomonas* [9, 14]. Transposases further contribute to the differences between *Endozoicomonas sensu stricto* and *Neoendozoicomonas*, with different types enriched in each of them. Finally, the signal proteins, ephrins, are found only in the genomes of *Neoendozoicomonas*, suggesting that their occurrence follows clade- or species-specific patterns [9]. Both pili and secretion systems provide mechanisms for bacterial adhesion to surfaces, facilitating host colonization [52], and these differences may therefore reflect genus-specific colonization strategies. Both genera encode a type III secretion system, commonly associated with host colonization by both mutualistic and opportunistic pathogens [53]. Lineages assigned to *Endozoicomonas sensu stricto* were observed carrying genes for effector apparatus linked to virulence [16], whereas *Neoendozoicomonas* spp. retains a more complete cluster for conjugative transfer (type IV secretion system components), despite potential lineage-specific losses of this machinery [16]. Additional traits highlighted by Gotze et al. [16] include divergences in carbohydrate metabolism, glucuronate metabolism, lipid degradation, genes for phosphorus acquisition, prevalence of genes for Type VI secretion system in *Endozoicomonas sensu stricto* and different mechanisms for cell evasion.

Our findings provide additional evidence of functional differentiation of these groups, particularly regarding the presence of conjugal transfer machinery (*tra* genes) in *Neoendozoicomonas*. Beyond these structural distinctions, our analysis suggests that metabolic traits may further differentiate these lineages. For instance, the enrichment of putrescine transport (*pot* genes) and *eutM* in *Endozoicomona*s indicates a potential capacity to assimilate specific nitrogen and carbon sources, such as holobiont-derived polyamines and phospholipids. Conversely, *Neoendozoicomonas* displayed enrichment in pathways for DNRA and *afuA*. The complete pathway for DNRA was previously noted in several genomes of *Endozoicomonas sensu stricto* [6], however, our finding showed that some *Endozoicomonas* lineages may have lost the nitrate reductase and kept only a nitrite reductase.

From a phenotypic point of view, Gotze et al. [17] evidenced that some lineages from *Endozoicomonas sensu stricto* form structured aggregates with clear boundaries by membrane formation, whilst lineages assigned to *Neoendozoicomonas* forms cell clusters lacking clear membrane-contained boundaries. Together, these findings imply that the divergence within the *Endozoicomonadaceae* may involve adaptations to distinct metabolic niches in addition to the variation in colonization factors described in prior studies.

The ecological and functional implications of these genomic traits remain challenging to interpret, particularly because the nature of *Endozoicomonas*–host interactions appears to vary across environmental settings. Although two *Endozoicomonas sensu stricto* species were reported as pathogenic in cobia and seabream fishes [10, 54], most isolates and metagenome-assembled genomes originate from invertebrate hosts, where no pathogenic role has been documented. Instead, several studies point toward potential contributions to nitrogen cycling and metabolic support [4, 6, 9, 14]. Given this diversity of contexts and outcomes, it seems unlikely that *Endozoicomonas sensu stricto* engages exclusively in a single type of interaction. Host association patterns instead appear to be shaped by lineage-specific traits and ecological context.

### Implications for the OGRIs framework

Our comparative assessment of OGRI metrics within the family *Endozoicomonadaceae* highlights cpAAI as a reliable metric for genus-level delimitation, with a recommended cutoff of 76% that aligns closely with phylogenomic relationships and preserves most currently accepted genera [40, 55]. In contrast, POCP failed to reveal clear breakpoints near the proposed 50% genus threshold [22] and exhibited high variability, while ANI also lacked resolution to separate genera, confirming its limited applicability at genus level [56]. At species level, however, ANI demonstrated robust clustering performance, with most species pairs having values >96%–98% identity, aligned with “standard” thresholds [56]. Our results support previous studies [57–59] arguing that AAI and cpAAI are more suitable metrics to define genus rank rather than ANI and POCP [Riesco & Trujillo 2024]. The breakpoint of 70% observed in AAI mostly resolved the genera clustering similarly to the breakpoint of 76% using cpAAI, indicating that AAI can serve as a reliable strategy. However, it is important to note that the breakpoint was more clearly defined using cpAAI than AAI, with a wider interval range. Therefore, we encourage the prioritization of cpAAI values as a robust proxy for genus delimitation in the *Endozoicomonadaceae* family to reduce potential artifacts, while also acknowledging AAI as an expedient alternative for analyses, particularly in overall comparative genomic studies and for fast results.

In summary, our genome-scale analyses resolve long-standing ambiguities in the taxonomy and evolutionary history of the *Endozoicomonadaceae* family, helping to bridge the gap between taxonomic nomenclature and metabolic functional potential. By formalizing the transition from informal “clades” to the erection of the distinct genera *Endozoicomonas sensu stricto* and *Neoendozoicomonas* gen. nov., we recognize the evolutionary distinctiveness which reflects different ecological strategies and host-interaction repertoires. In the context of accelerating global change and rapid restructuring of marine ecosystems, distinguishing between different strategies becomes increasingly important. Our findings suggest that *Endozoicomonas sensu stricto* may preferentially adopt a strategy centered on active host-surface colonization and metabolic flexibility, whereas *Neoendozoicomonas* appears more specialized toward distinct metabolic niches and potentially more intimate host associations. In parallel, the characterization of *Endonucleibacter* and *Sororendozoicomonas* as genomically streamlined lineages highlights a broad spectrum of symbiotic host dependence within the family *Endozoicomonadaceae*, ranging from versatile associates to host-restricted specialists. Importantly, this refined taxonomic framework enables improved discrimination of *Endozoicomonadaceae* lineages in commonly applied approaches such as 16S rRNA gene–based surveys, which have historically masked this diversity. Beyond its taxonomic implications, the proposed 76% cpAAI threshold provides a standardized framework for linking phylogenetic structure to ecological function, thereby further improving our capacity to understand the roles of members of the family *Endozoicomonadaceae* in host health, nutrient cycling, and holobiont resilience. By refining taxonomic resolution at the genus level, this framework enables more accurate taxonomic and ecological inference and lays the groundwork for future efforts aimed at understanding,—and potentially leveraging—the functional diversity of these ubiquitous symbionts in a changing ocean.

### Genome-based description of the genera on Endozoicomonadaceae family

- **Emended description of the genus *Endozoicomonas***

Name description: En.do.zo.i.co’mo.nas. Gr. pref. *endo*-, inside; Gr. masc. adj. zôïkos, animal; L. fem. n. *monas*, monad; N.L. fem. n. *Endozoicomonas*, a monad living inside an animal (https://lpsn.dsmz.de/genus/endozoicomonas) [1].

Firstly described by Kurahashi and Yokota [1] (2007), *E. elysicola* was isolated from the sea slug *Elysia ornata*. The type species for this genus is *E. elysicola*. The GC content of the genus is 48.26% (± 2.48), with an average genome size of 5.60 Mbp (± 0.96).

The current species list include *E. acroporae* (Sheu et al. 2017) [60], *E. arenosclerae* (Appolinario et al. 2016) [61], *E. ascidiicola* (Schreiber et al. 2016) [62], *E. atrinae* (Hyun et al. 2014) [63], *E. coralli* (Chen et al. 2020) [64], *E. elysicola* (Kurahashi and Yokota 2007) [1], *E. euniceicola* (Pike et al. 2013) [65], *E. gorgoniicola* (Pike et al. 2013) [65], *E. lisbonensis* (da Silva et al. 2025) [7], *E. montiporae* (Yang et al. 2010) [46], *E. numazuensis* (Nishijima et al. 2013) [2], *E. promiscua* (Modolon et al. 2025) [6], *E. marisrubri* (Pogoreutz et al. 2022) [14]. In addition, species within prefix *Candidatus* include *Candidatus* E. cretensis (Katharios et al. 2015) [10], *Candidatus* E. endoleachii (Hyams et al. 2023) [66], *Candidatus* E. penghunesis (Tandon et al. 2022) [67] and *Candidatus* E. ruthgatesiae (Chiou et al. 2023) [68]. The proposed species to keep with current nomenclature, in the genus *Endozoicomonas*, include: *E. acroporae* (seqco.de/i:7065); *E. ascidiicola* (seqco.de/i:7067); *E. atrinae* (seqco.de/i:7068); *E. coralli* (seqco.de/i:7069); *E. elysicola* (seqco.de/i:7070); *E. promiscua* (seqco.de/i:49122), *Candidatus* E. cretensis (no SeqCode entry).

- **Description of the *Neoendozoicomonas* gen. nov.**

Name description: Ne.o.en.do.zo.i.co’mo.nas. Gr. prep. *Neo*, new; N.L.fem. n. *Endozoicomonas*, a bacterial genus; N.L. fem. n. *Neoendozoicomonas*, new genus *Endozoicomonas.*

Proposed species: *Neoendozoicomonas montiporae* comb. nov.; *N. euniceicola* comb. nov.; *N. gorgoniicola* comb. nov.; *N. lisbonensis* comb. nov.; *N. arenosclerae* comb. nov.; *N. numazensis* comb. nov.;

Members of the genus are Gram-stain-negative, non-spore-forming, and primarily associated with marine invertebrate hosts. Members of this genus possess comparatively large genomes within the family *Endozoicomonadaceae*, with an average genome size of 6.22 Mbp (± 0.85) and a mean GC content of 47.92% (± 0.91). Genomes assigned to *Neoendozoicomonas* show high coherence in protein sequences, sharing >70% AAI and > 76% core-protein AAI (cpAAI), forming a well-supported monophyletic group distinct from *Endozoicomonas sensu stricto* and from the streamlined genera *Endonucleibacter* and *Sororendozoicomonas*. The genus *Neoendozoicomonas* is distinguished from the genus *Endozoicomonas* by an expanded repertoire of genes associated with conjugative transfer and genome plasticity, including a conserved cluster of TraABCDEFIKLNUV proteins. Additional lineage-specific enrichments include genes involved in carbohydrate uptake and central carbon metabolism (such as two *glk* orthologs, PTS-related components, and glycerol-3-phosphate transporters), as well as determinants for lipid modification and membrane remodeling (e.g. *lpxP*, acyl-CoA thioester hydrolase). Some genomes also encode unique regulatory and stress-response features (e.g. MarR-family regulators, RfaH, and fluoroquinolone resistance genes).

The type species of this genus is *Neoendozoicomonas montiporae* (Yang et al. 2010) [46]*.*

- *Neoendozoicomonas montiporae* (Yang et al. 2010) comb. nov.

Name description: N.L. gen. fem. n. *montiporae*, of Montipora, referring to the isolation of the type strain from a coral belonging to the genus Montipora.

Basonym: *E. montiporae* Yang et al. 2010 (SeqCode Registry: seqco.de/i:7073).

Type: Strain CL-33 (=BCRC 17933 = LMG 24815).

Description: As described in the original species description [46].

- *Neoendozoicomonas euniceicola* (Pike et al. 2013) comb. nov.

Name description: N.L. fem. n. *Eunicea*, name of a zoological genus; L. masc./fem. n. suff. *-cola*, dweller; N.L. masc./fem. n. *euniceicola*, Eunicea dweller.

Basonym: *Endozoicomonas euniceicola* Pike et al. 2013 (SeqCode Registry: seqco.de/i:7071).

Type: Strain EF212 (=DSM 26535 = NCCB 100458).

Description: As described in the original species description [65].

- *Neoendozoicomonas gorgoniicola* (Pike et al. 2013) comb. nov.

Name description: Gr. fem. n. *Gorgonia*, name of a zoological genus; L. masc./fem. n. suff. *-cola*, dweller; N.L. masc./fem. n. *gorgoniicola*, Gorgonia dweller.

Basonym: *Endozoicomonas gorgoniicola* Pike et al. 2013 (SeqCode Registry: seqco.de/i:7072).

Type: Strain PS125 (=DSM 26534 = CECT 8353 = NCCB 100438).

Description: As described in the original species description [65].

- *Neoendozoicomonas lisbonensis* (da Silva et al. 2025) comb. nov.

Name description: N.L. fem. adj. lisbonensis, pertaining to Lisbon, Portugal.

Basonym: *Endozoicomonas lisbonensis* da Silva et al. 2025 (SeqCode Registry: seqco.de/i:50032).

Type: Strain NE40 (=DSM 118084 = UCCCB 212).

Description: As described in the original species description [7].

- *Neoendozoicomonas arenosclerae* (Appolinario et al. 2016) comb. nov.

Name description: N.L. gen. fem. n. arenosclerae, of the sponge *Arenosclera brasiliensis*.

Basonym: *Endozoicomonas arenosclerae* Appolinario et al. 2016 (SeqCode Registry: seqco.de/i:7066).

Type: Strain ab112 (=CBAS 572 = NBRC 108893).

Description: As described in the original species description [61].

- *Neoendozoicomonas numazuensis* (Nishijima et al. 2013) comb. nov.

Name description: N.L. fem. adj. numazuensis, of or pertaining to Numazu, Japan.

Basonym: *Endozoicomonas numazuensis* Nishijima et al. 2013 (SeqCode Registry: seqco.de/i:7074).

Type: Strain HC50 (=DSM 25634 = KCTC 32099^T^).

Description: As described in the original species description [2].

- *Neoendozoicomonas marisrubri* (Pogoreutz et al. 2022) comb. nov.

Name description: L. neut. n. *mare* (gen. maris), the sea; L. masc. adj. *ruber*, red; N.L. gen. neut. n. *marisrubri*, of the Red Sea.

Basonym: *Endozoicomonas marisrubri* Pogoreutz et al. 2022 (not currently in SeqCode).

Type: Strain 6c.

Description: As described in the original species description [14].

- *Neoendozoicomonas ruthgatesiae* (Chiou et al. 2023) comb. nov.

Name description: N.L. fem. n. *ruthgatesiae*, of Ruth Gates, a pioneer in coral reef research.

Basonym: *Candidatus Endozoicomonas ruthgatesiae* Chiou et al. 2023 (not currently in SeqCode).

Type: Strain 8E.

Description: As described in the original species description [68].

- *Neoendozoicomonas penghunensis* (Tandon et al. 2022) comb. nov.

Name description: N.L. fem. adj. *penghunensis*, pertaining to the Penghu Archipelago, Taiwan.

Basonym: *Candidatus Endozoicomonas penghunensis* Tandon et al. 2022 (not currently in SeqCode).

Type: Strain 4G.

Description: As described in the original species description [67].

- **Emended description of the genus *Sansalvadorimonas* Goldberg et al. 2018**

*Sansalvadorimonas* (San.sal.va.do.ri.mo’nas. N.L. fem. n. *Sansalvadorimonas*, a monad from San Salvador).

The description is as provided by Goldberg et al. [69] with the following emendations. The genus is defined genomically by a cpAAI >76% and forms a distinct monophyletic clade in phylogenomic reconstructions. Members are Gram-stain-negative, marine-associated bacteria isolated from sponges and other invertebrates.

Nomenclatural note: Following the principle of priority (ICNP; SeqCode Rule 23d), *Sansalvadorimonas* (validly published June 2018) is the senior synonym of *Parendozoicomonas* (validly published July 2018). This emendation unites the two genera, rectifying the dubious status of *Sansalvadorimonas* created in previous revisions (Kim et al. 2024) and correctly transferring species formerly assigned to *Parendozoicomonas* to this genus.

The type species is *Sansalvadorimonas verongulae* (SeqCode Registry: seqco.de/i:18727).

- *Sansalvadorimonas haliclonae* (Bartz et al. 2018) comb. nov.

Name description: N.L. gen. n. *haliclonae*, of *Haliclona*, a genus of marine sponge.

Basonym: *Parendozoicomonas haliclonae* Bartz et al. 2018 (SeqCode Registry: seqco.de/i:15488).

Type: Strain S-B4-1U (=CCM 8713 = DSM 10367 = LMG 29769).

Description: As described in the original species description [70].

- *Sansalvadorimonas callyspongiae* (Kim et al. 2024) comb. nov.

Name description: N.L. gen. n. *callyspongiae*, of *Callyspongia*, a genus of marine sponge.

Basonym: *Parendozoicomonas callyspongiae* Kim et al. (2024) (SeqCode Registry: seqco.de/i:46086).

Type: Strain 2012CJ34–2 (= KACC 22641 = LMG 32581).

Description: As described in the original species description [50].

## Supplementary Material

Supplementary_materials_ycag123

## Data Availability

All data analyzed during this study are included in this published article and its supplementary information files. All genome assemblies referenced are publicly available via the NCBI database (accession numbers provided in Supplementary Table S1). Furthermore, all scripts, software dependencies, figure reproduction codes, 16S rRNA gene sequences, and concatenated orthologous clusters used for phylogenomics inferences are available in the following repository: https://github.com/modolon/phylo_endozoi.
