# Supplementary material for: Resolving the evolutionary duality of marine symbionts: redefining the genus Endozoicomonas and proposing Neoendozoicomonas gen. nov
Source: ISME Commun. 2026 May 13;6(1):ycag123. doi: 10.1093/ismeco/ycag123 (PMC13286007; doi:10.1093/ismeco/ycag123)
Supplement: Supplementary_materials_ycag123 [file supplementary_materials_ycag123.zip › Supp_Info_final_Modolon_et_al_April_26_Endozoico.docx]

**Resolving the evolutionary duality of marine symbionts: Redefining the genus *Endozoicomonas* and proposing *Neoendozoicomonas* gen. nov.**

Fluvio Modolon¹*, Alessandro N. Garritano^2,3^, Philip Hugenholtz^4^, Christian R. Voolstra^5^, Tina Keller-Costa^6,7^, Rodrigo Costa^6,7^, Torsten Thomas^3^, Raquel S. Peixoto^8^*

**Affiliations**

¹Department of Ecology, Environment and Geoscience, Umeå University, Umeå, Sweden;

^2^School of Life and Environmental Sciences, University of Sydney, Australia;

^3^Centre for Marine Science and Innovation & School of Biological, Earth and Environmental Sciences, The University of New South Wales, Sydney, NSW 2052, Australia;

^4^Australian Centre for Ecogenomics, School of Chemistry and Molecular Biosciences, The University of Queensland, Australia;

^5^Department of Biology, University of Konstanz, Konstanz, Germany;

^6^Institute for Bioengineering and Biosciences (iBB) and Institute for Health and Bioeconomy (i4HB), Instituto Superior Técnico, University of Lisbon, Lisbon, Portugal;

^7^Department of Bioengineering, Instituto Superior Técnico, University of Lisbon, Lisbon, Portugal;

^8^Biological and Environmental Science and Engineering Division, King Abdullah University of Science and Technology, Thuwal, 23955, Saudi Arabia

***Corresponding authors**

Fluvio Modolon - [fluvio.modolon@umu.se](mailto:fluvio.modolon@umu.se)

Raquel S. Peixoto - [raquel.peixoto@kaust.edu.sa](mailto:raquel.peixoto@kaust.edu.sa)

| **Comparative Orthogroup Analysis** | **Count** | **Average** |
| --- | --- | --- |
| **Total orthogroups in target genera:** | 13731 | NA |
| **Core orthogroups (all three genera):** | 2985 | NA |
|  |  |  |
| **Exclusive Orthogroups per Genus** | | |
| *Endonucleibacter* (6 genomes): | | |
| - Total exclusive OGs: | 984 | 164 |
| - 100% conserved (all genomes): | 14 | NA |
| - 75% conserved (≥5 genomes): | 20 | NA |
| - 50% conserved (≥3 genomes): | 88 | NA |
|  |  |  |
| *Endozoicomonas* (18 genomes): | | |
| - Total exclusive OGs: | 2945 | 163.6 |
| - 100% conserved (all genomes): | 13 | NA |
| - 75% conserved (≥10 genomes): | 83 | NA |
| - 50% conserved (≥7 genomes): | 341 | NA |
|  |  |  |
| *Neoendozoicomonas* (23 genomes): | | |
| - Total exclusive OGs: | 3794 | 172.45 |
| - 100% conserved (all genomes): | 14 | NA |
| - 75% conserved (≥11 genomes): | 74 | NA |
| - 50% conserved (≥7 genomes): | 326 | NA |
|  |  |  |
| **Shared Orthogroups between Genus Pairs** | | |
| - *Endonucleibacter* & *Endozoicomonas*: | 334 | NA |
| - *Endonucleibacter* & *Neoendozoicomonas*: | 371 | NA |
| - *Endozoicomonas* & *Neoendozoicomonas*: | 2318 | NA |
|  |  |  |
| **Singleton Gene Counts per Genus** | | |
| - *Endonucleibacter*: | 1441 | 240.2 |
| - *Endozoicomonas*: | 4879 | 271 |
| - *Neoendozoicomonas*: | 3504 | 159.3 |

**Table S1.** Summarized distribution of orthologous gene clusters (orthogroups) across *Endonucleibacter*, *Endozoicomonas*, and *Neoendozoicomonas*, as inferred by OrthoFinder. Total orthogroups represent shared evolutionary units across all genera, while exclusive orthogroups reflect lineage-specific gene sets with varying conservation thresholds (full, partial, or minimal representation within a genus). Shared orthogroups highlight functional overlaps between genera, including a core set conserved in all three lineages. Singleton genes, excluded from orthogroups, indicate rare or unique genomic traits.


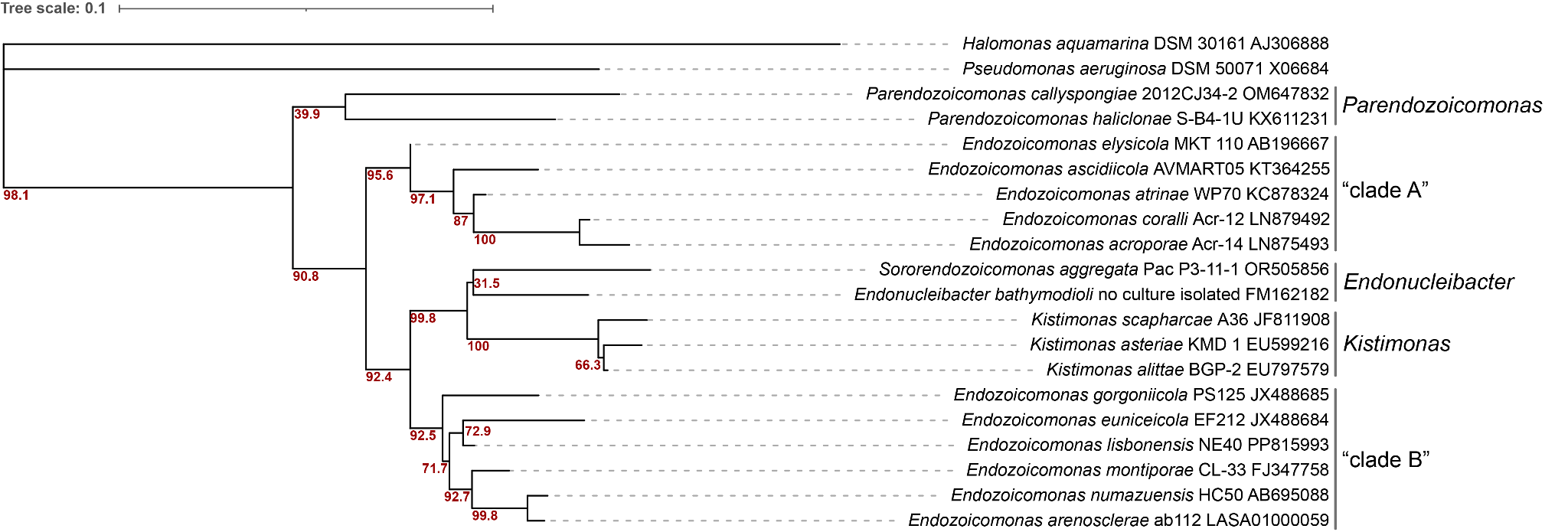


**Figure S1**. Phylogenetic tree based on rRNA 16S gene partial sequence. The tree scale is shown in the upper left corner. Red numbers indicate the bootstrap support (1,000 replicates).


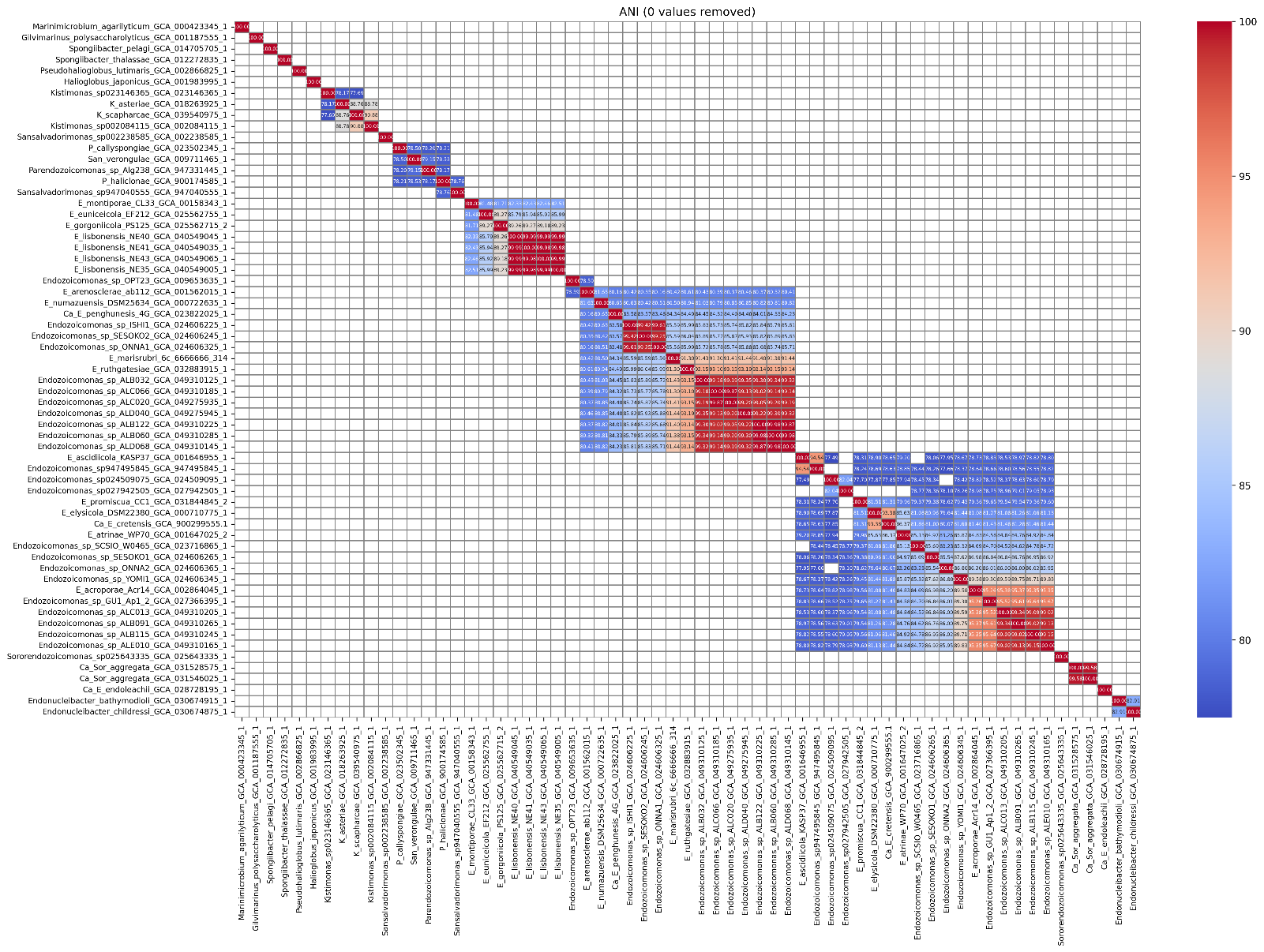


**Figure S2.** Heatmap for ANI showing the pairwise results. Values down below 77% were automatically removed by the fastani pipeline due to low confidential results.


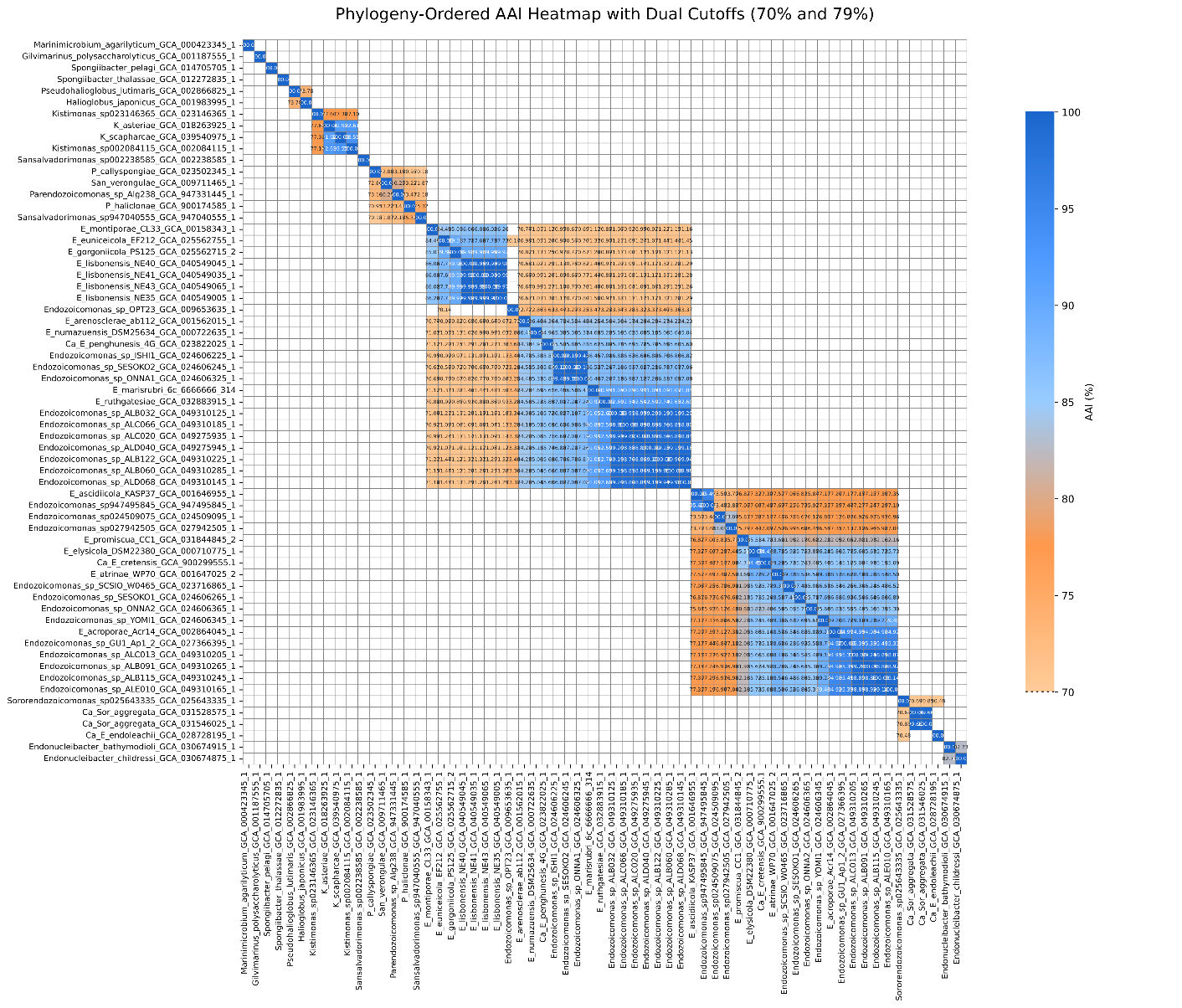


**Figure S3.** Heatmap for AAI applying 70% cutoff.

**
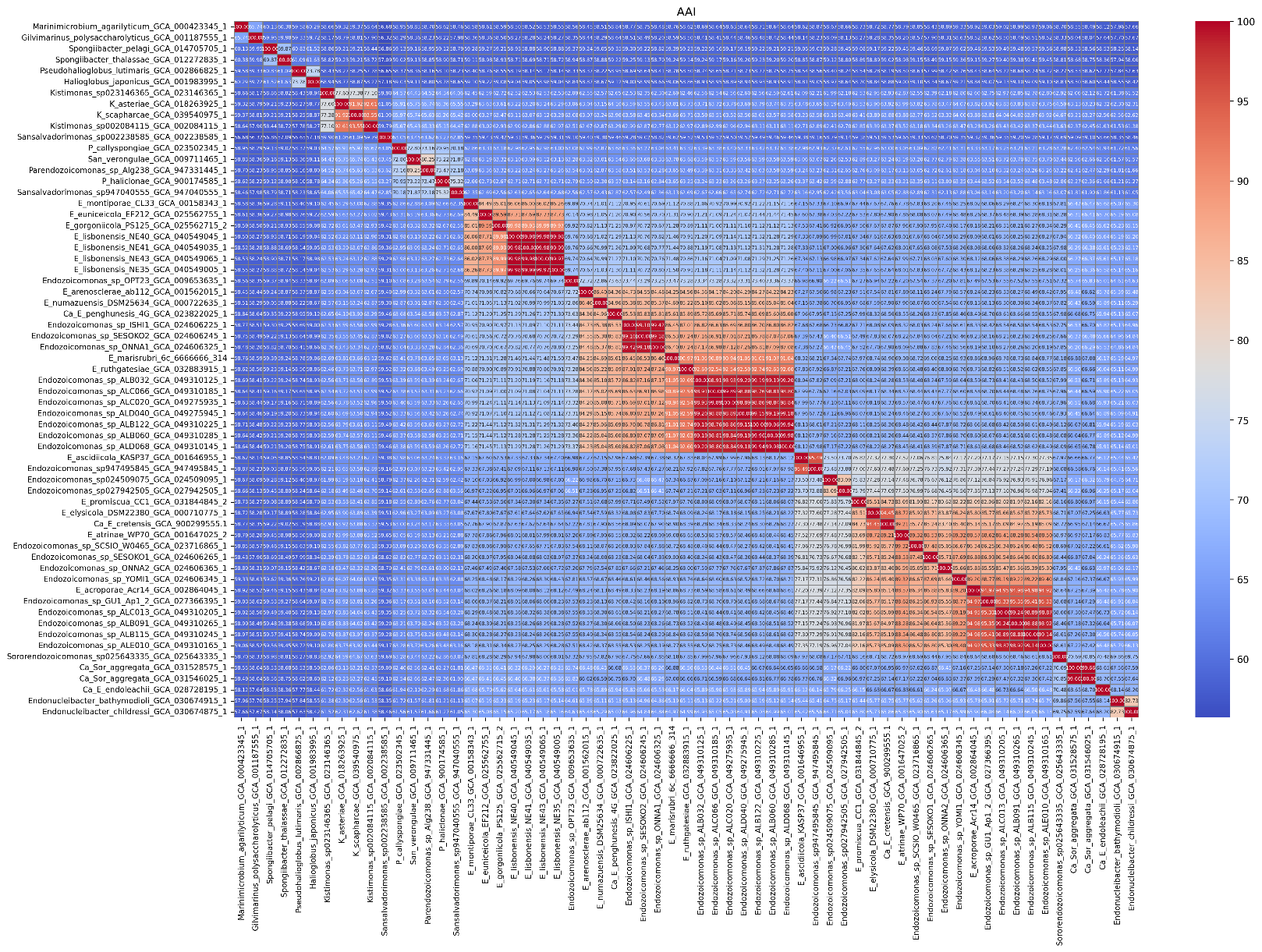
**

**Figure S4.** Heatmap for AAI showing the full range of values.


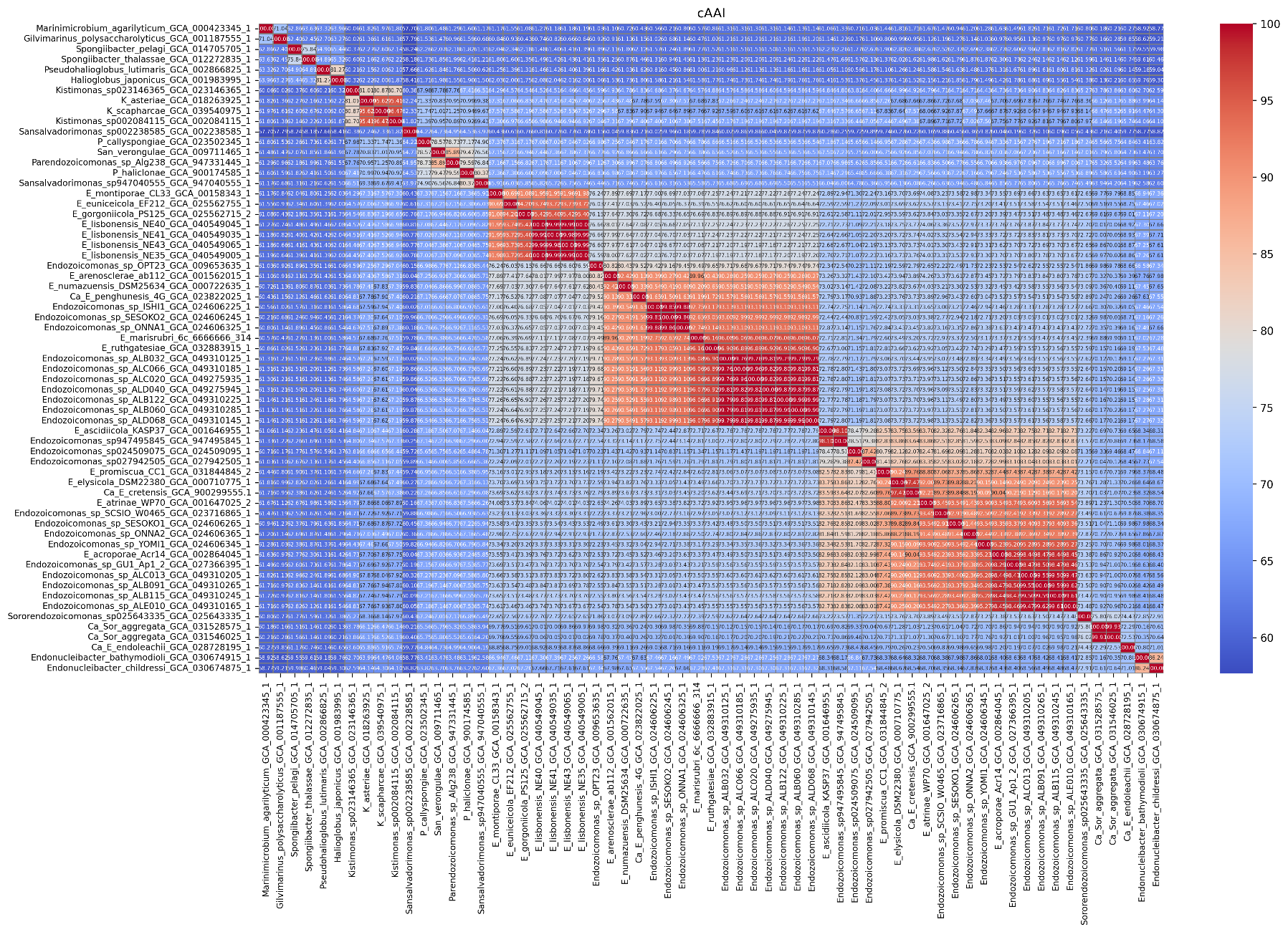


**Figure S5.** Heatmap for cpAAI showing the full range of values.

**
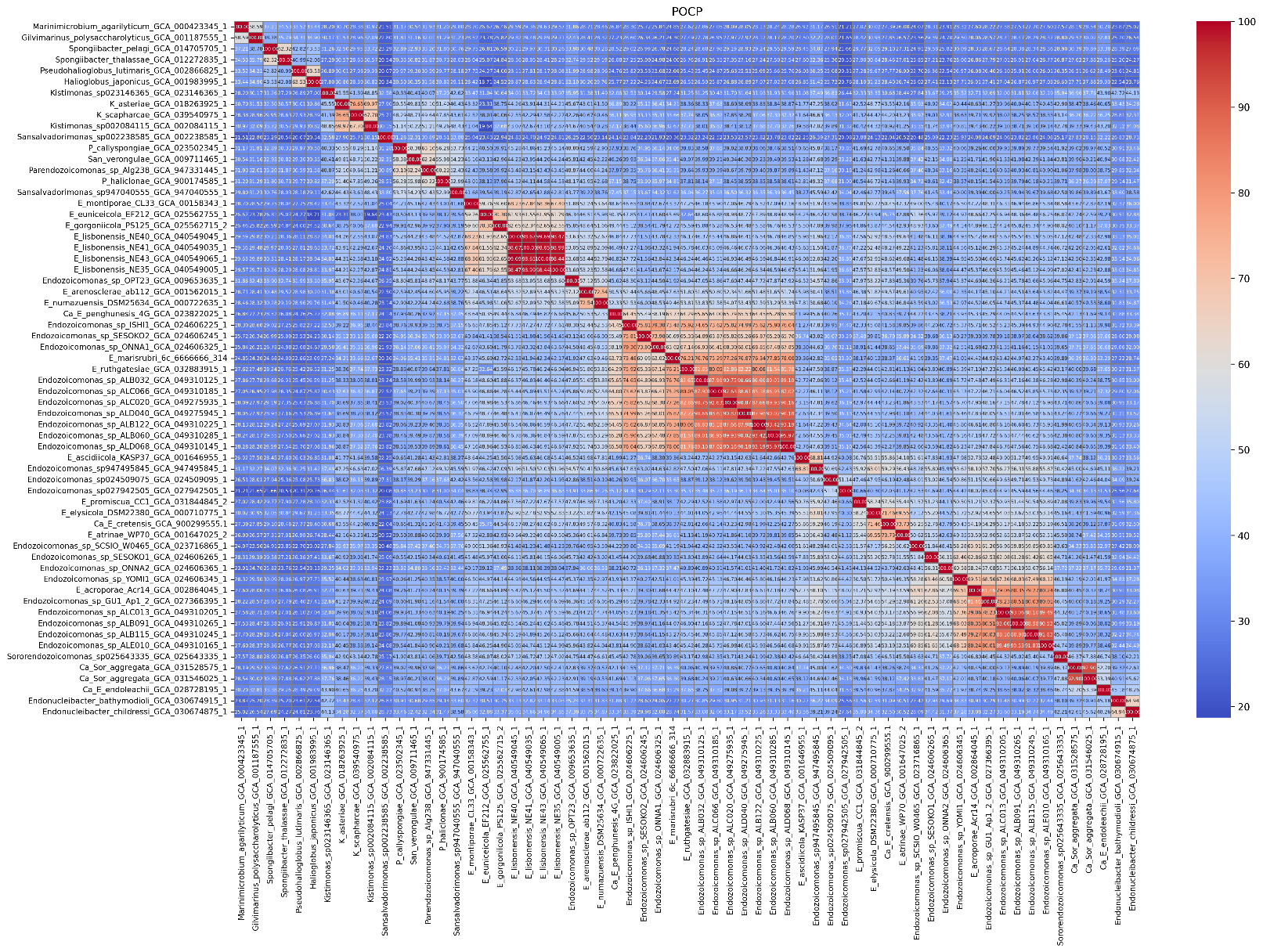
**

**Figure S6.** Heatmap for POCP showing the full range of values.

**
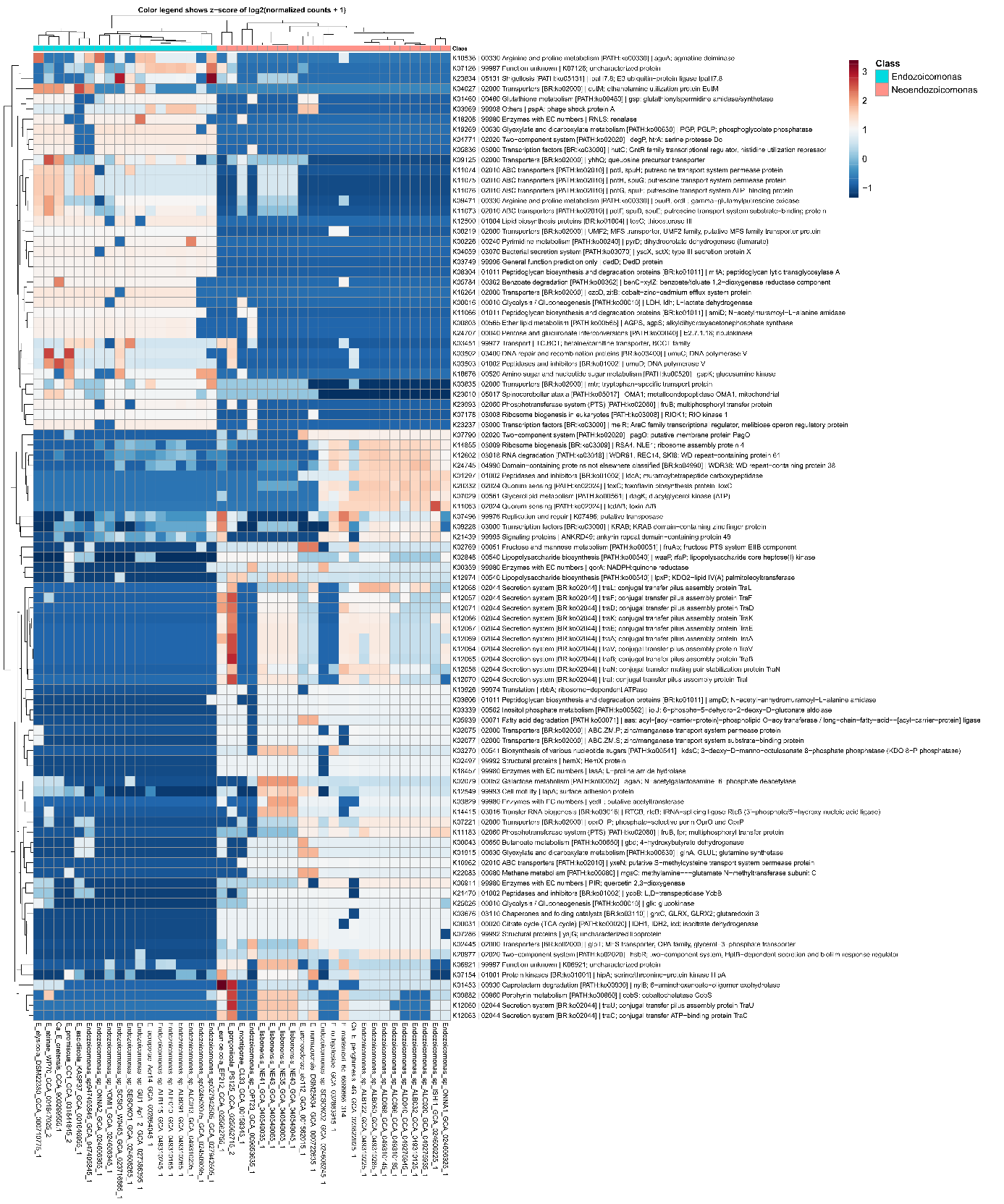
**

**Figure S7**. Heatmap of significant KEGG orthologs from differential functional abundance between *Endozoicomonas* and *Neoendozoicomonas*. Heatmap of z-scores for log₂-transformed normalized counts of KEGG orthologs significantly differing among the different genera. Rows show orthologs with functional annotations, and columns represent individual genomes. Color gradients indicate relative abundance levels, from low (blue) to high (red).


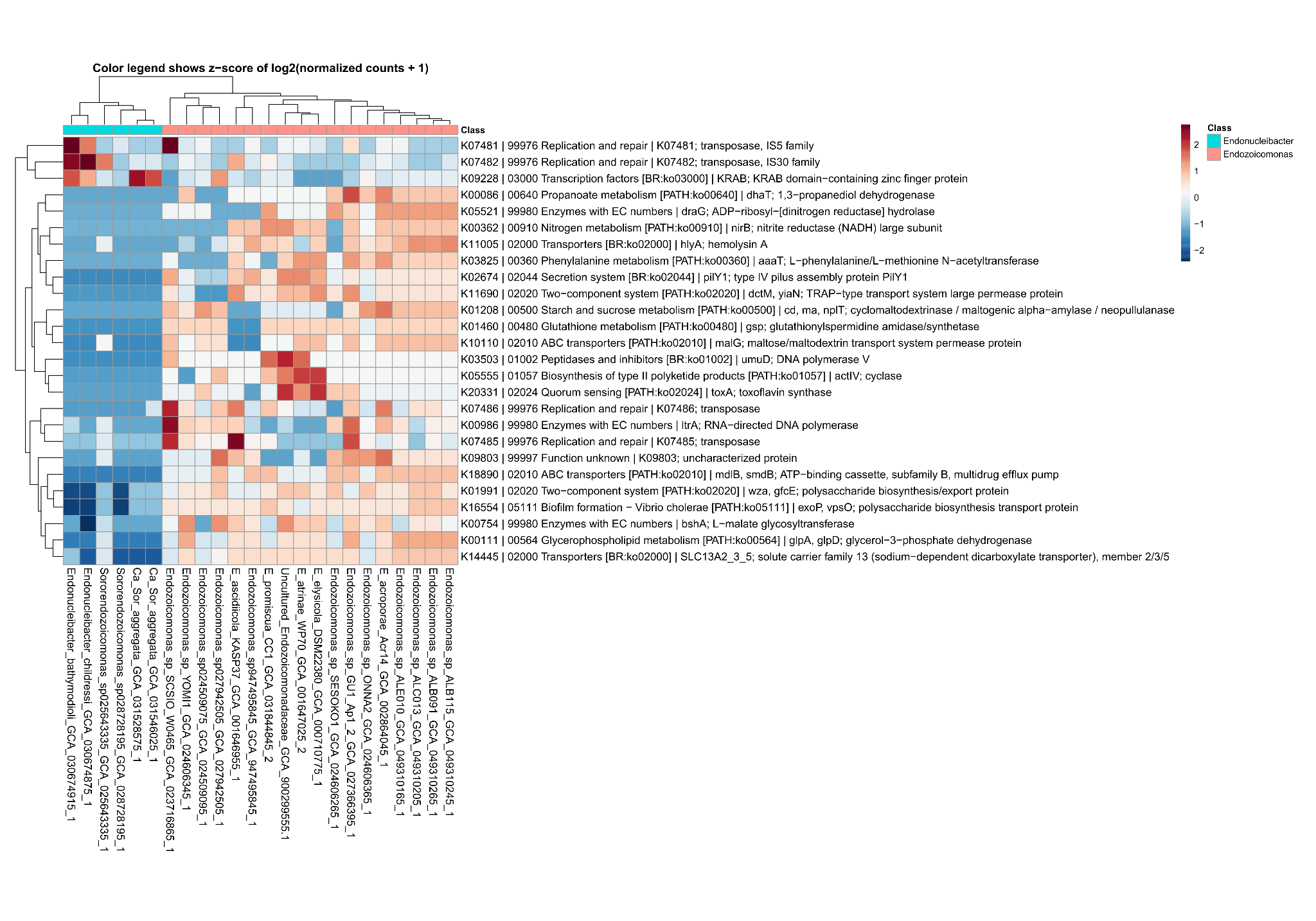


**Figure S8**. Heatmap of significant KEGG orthologs from differential functional abundance between *Endozoicomonas* and *Endonucleibacter/Sororendozoicmonas*. Heatmap of z-scores for log₂-transformed normalized counts of KEGG orthologs significantly differing among the different genera. Rows show orthologs with functional annotations, and columns represent individual genomes. Color gradients indicate relative abundance levels, from low (blue) to high (red).


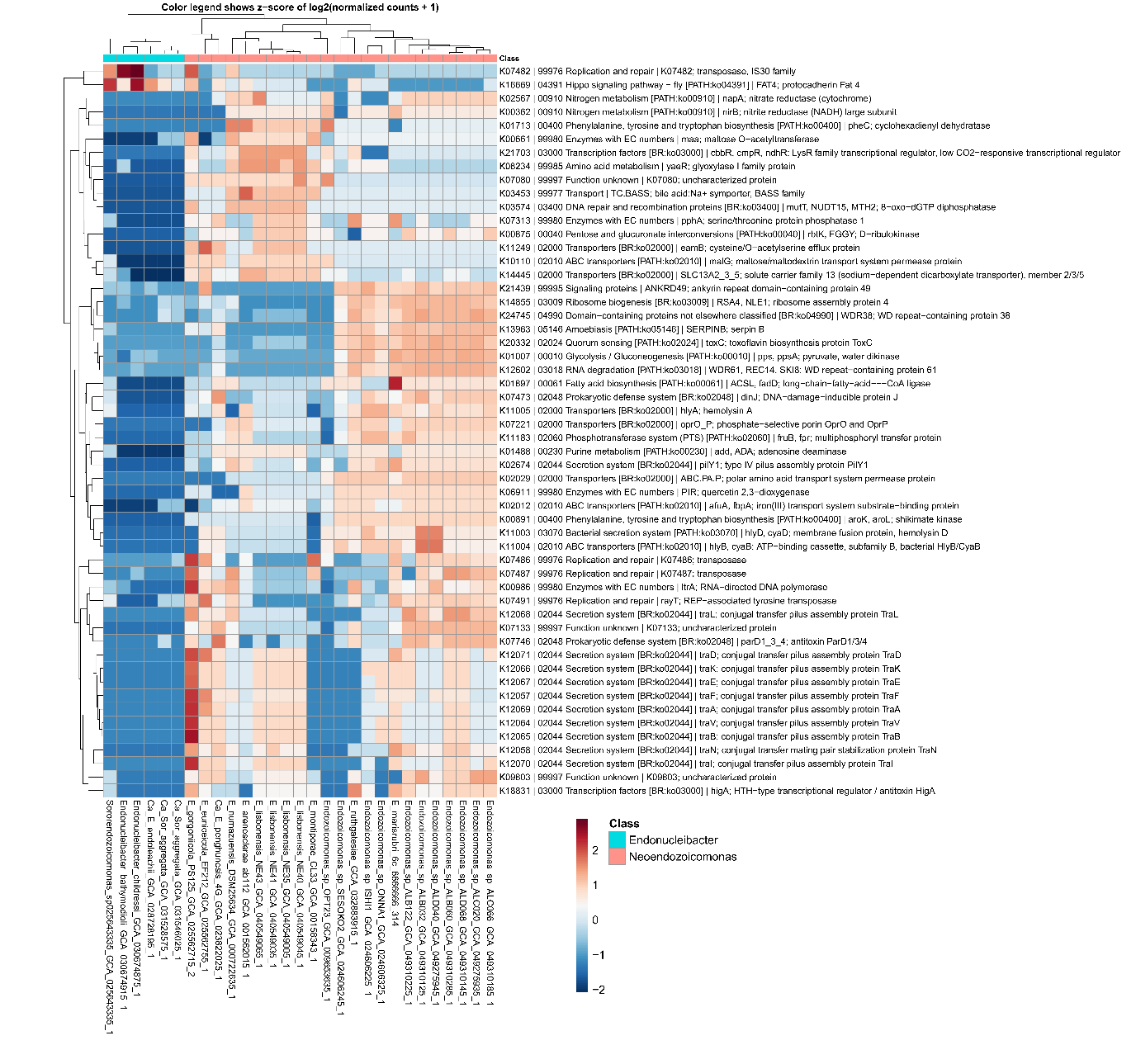


**Figure S9**. Heatmap of significant KEGG orthologs from differential functional abundance between *Endonucleibacter* and *Neoendozoicomonas*. Heatmap of z-scores for log₂-transformed normalized counts of KEGG orthologs significantly differing among the different genera. Rows show orthologs with functional annotations, and columns represent individual genomes. Color gradients indicate relative abundance levels, from low (blue) to high (red).
