# Supplementary figures and images for: Resolving the evolutionary duality of marine symbionts: redefining the genus Endozoicomonas and proposing Neoendozoicomonas gen. nov
Source: ISME Commun. 2026 May 13;6(1):ycag123. doi: 10.1093/ismeco/ycag123 (PMC13286007; doi:10.1093/ismeco/ycag123)

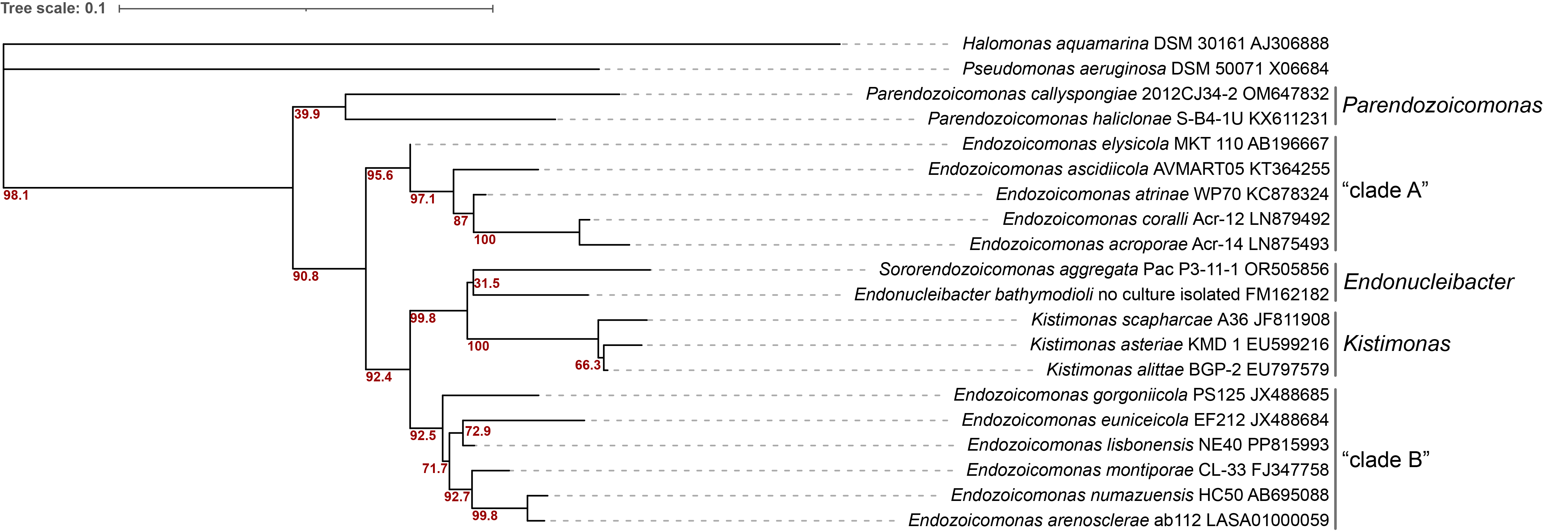

Supplement: Supplementary_materials_ycag123 [file supplementary_materials_ycag123.zip › FigS1.png]

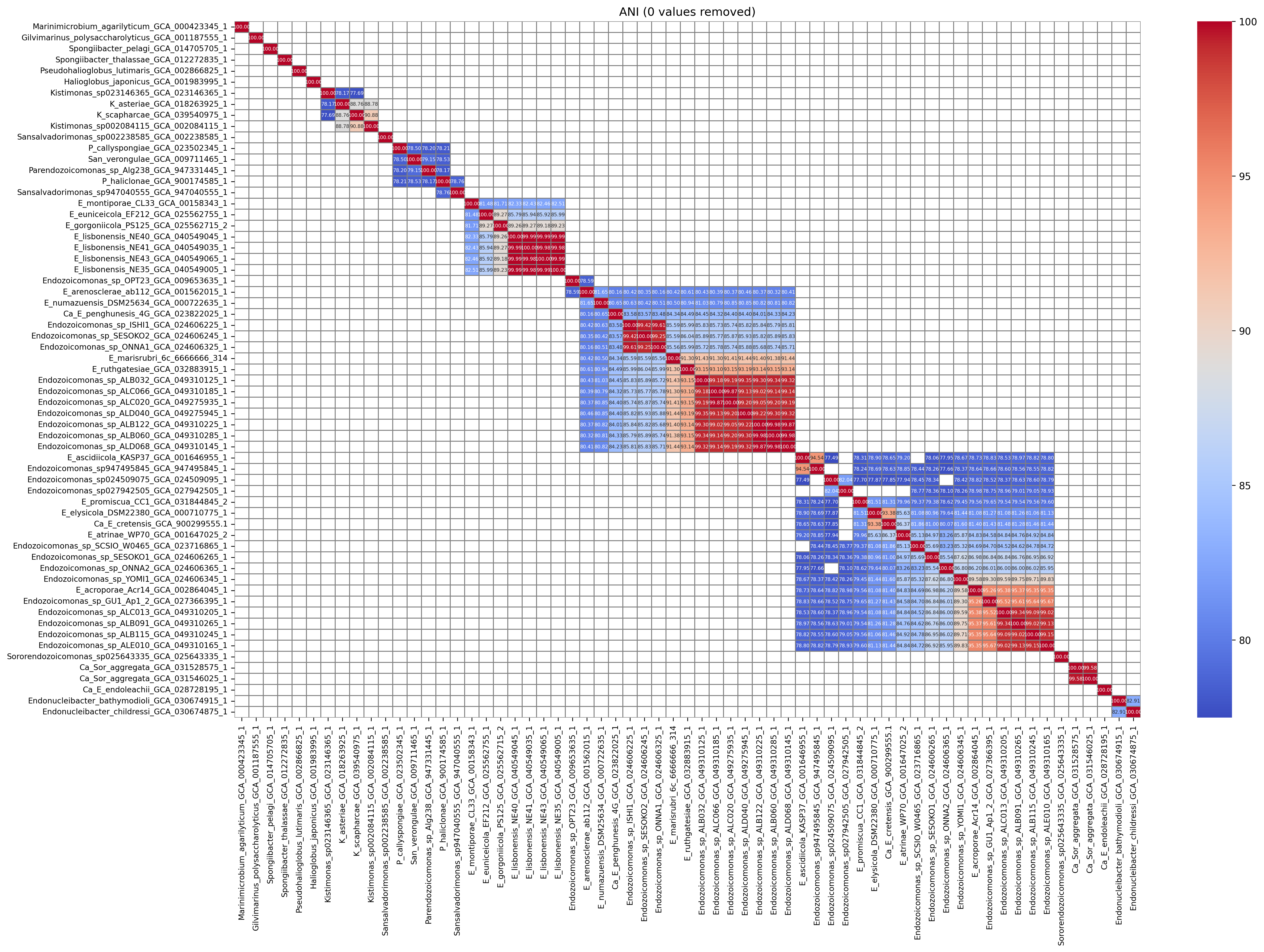

Supplement: Supplementary_materials_ycag123 [file supplementary_materials_ycag123.zip › FigS2.png]

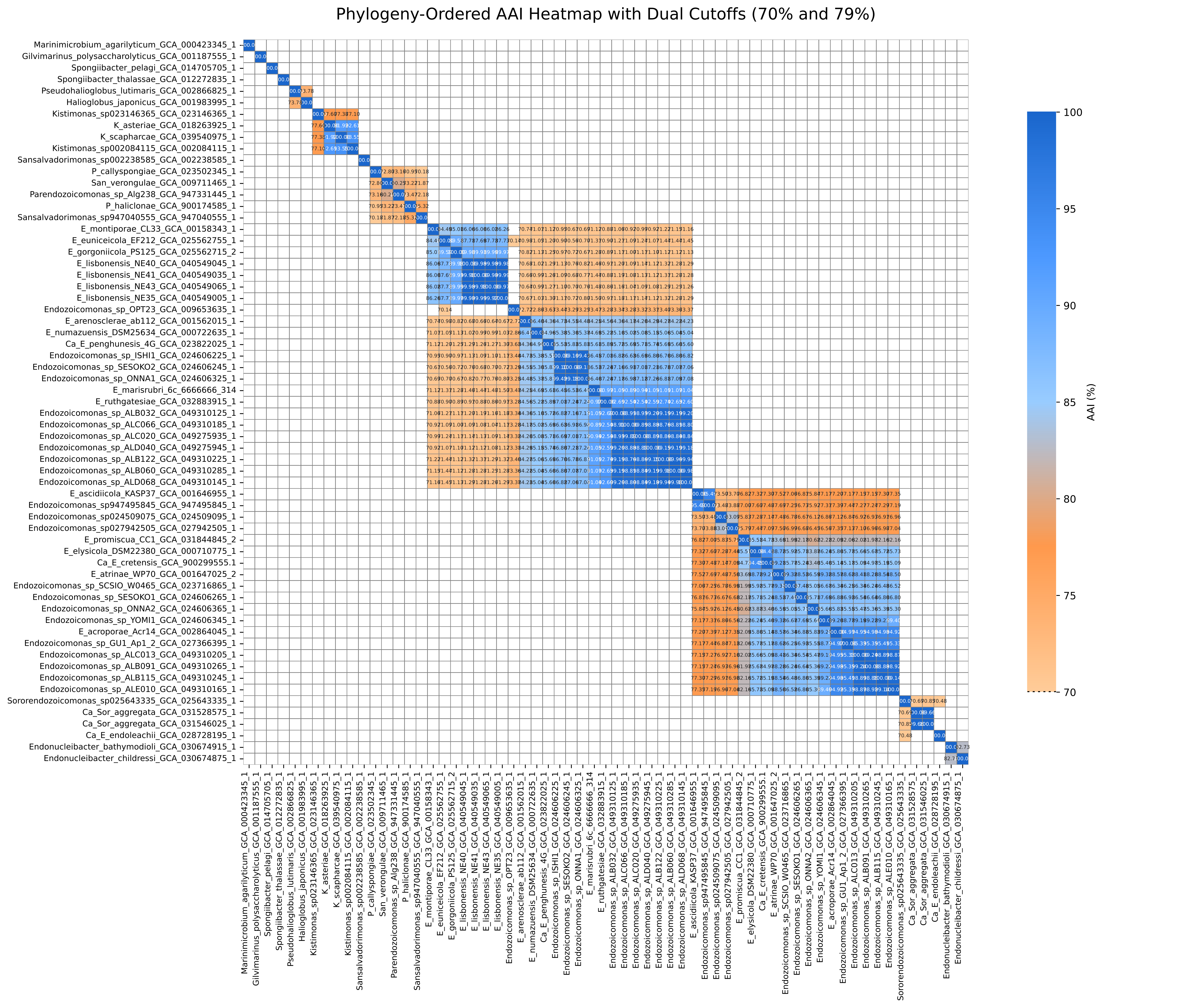

Supplement: Supplementary_materials_ycag123 [file supplementary_materials_ycag123.zip › FigS3.png]

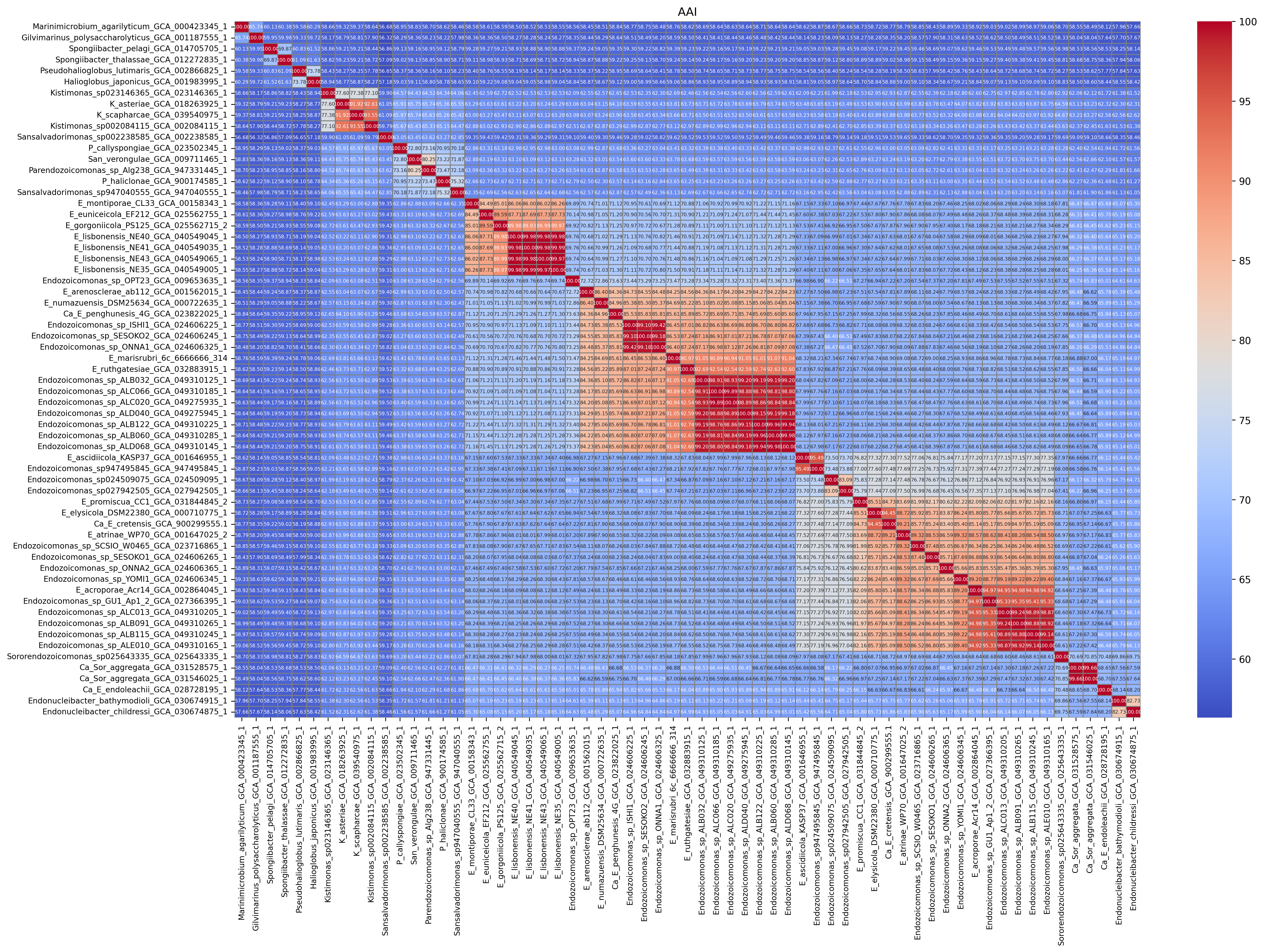

Supplement: Supplementary_materials_ycag123 [file supplementary_materials_ycag123.zip › FigS4.png]

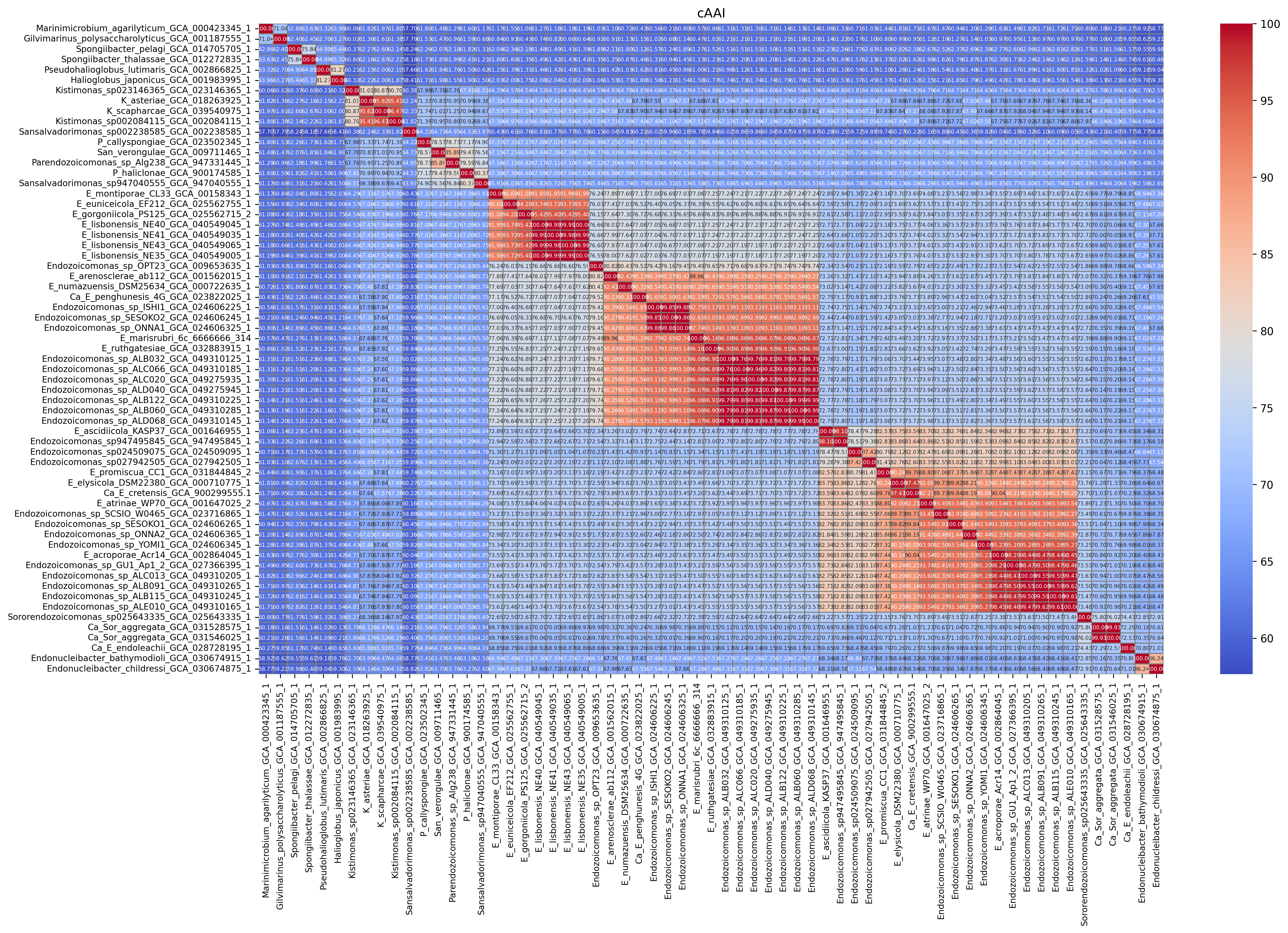

Supplement: Supplementary_materials_ycag123 [file supplementary_materials_ycag123.zip › FigS5.png]

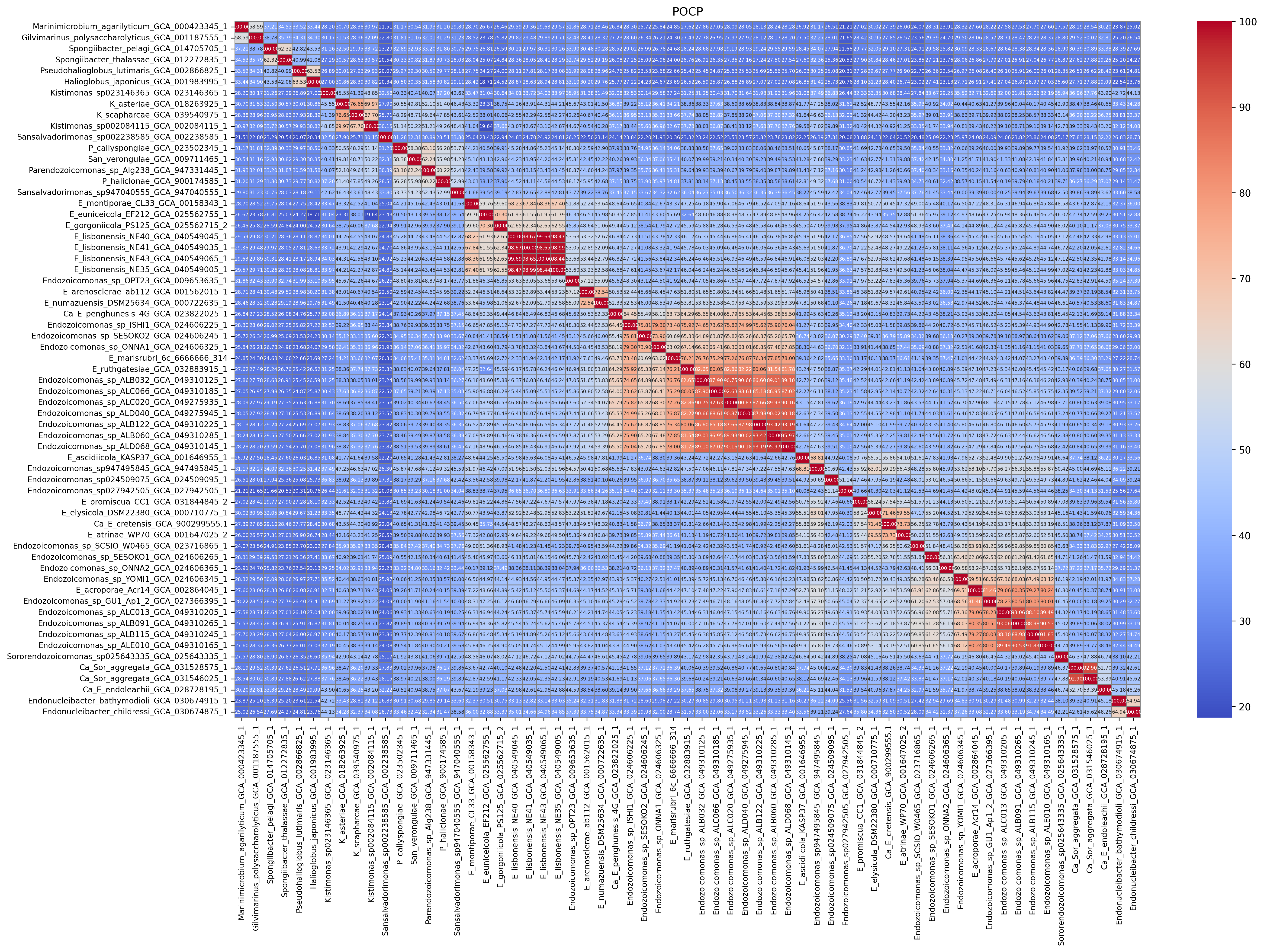

Supplement: Supplementary_materials_ycag123 [file supplementary_materials_ycag123.zip › FigS6.png]

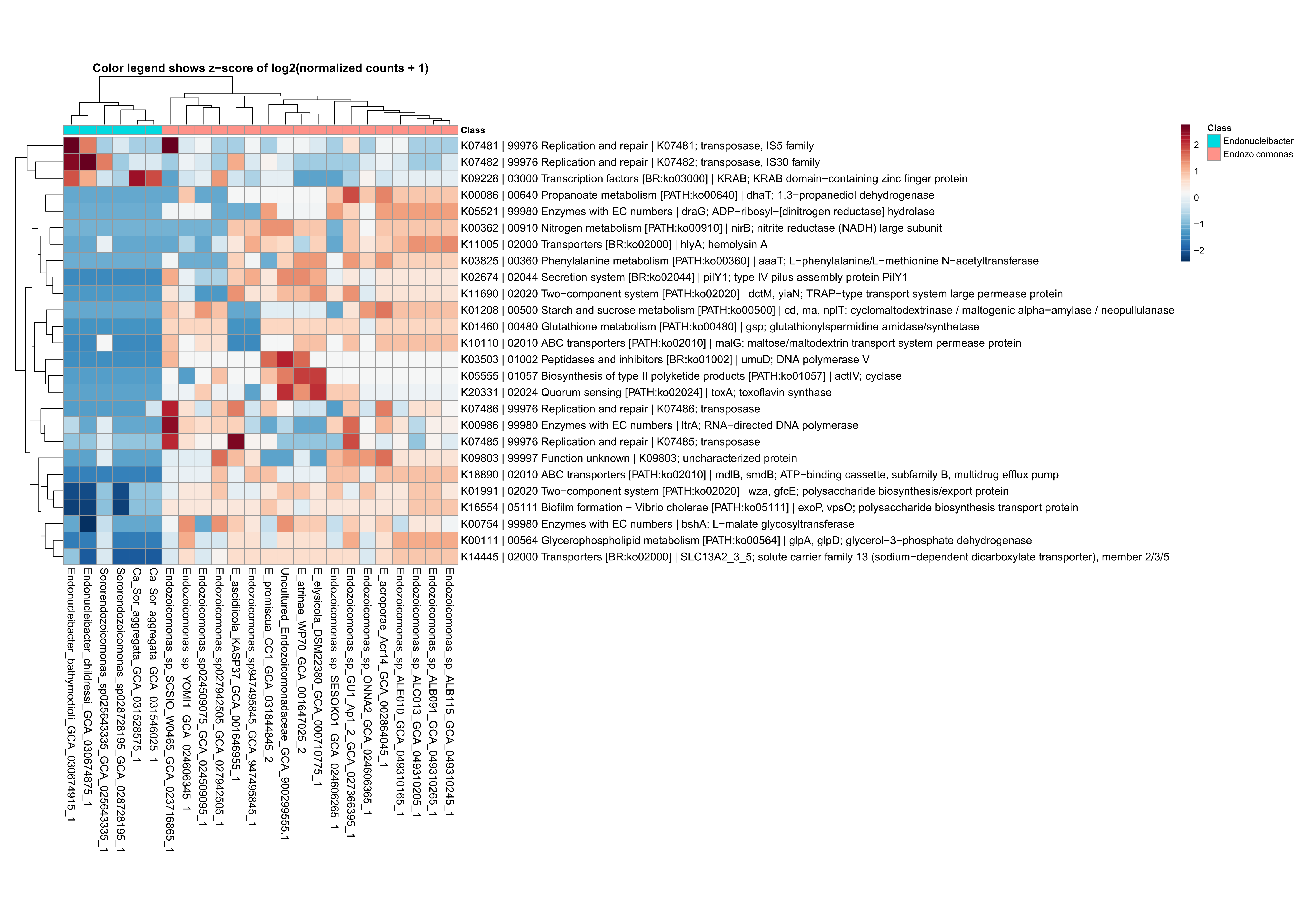

Supplement: Supplementary_materials_ycag123 [file supplementary_materials_ycag123.zip › FigS8.png]
